# Filamin A cooperates with the androgen receptor in preventing skeletal muscle senescence

**DOI:** 10.1038/s41420-023-01737-y

**Published:** 2023-12-02

**Authors:** Marzia Di Donato, Antimo Moretti, Carmela Sorrentino, Giuseppe Toro, Giulia Gentile, Giovanni Iolascon, Gabriella Castoria, Antimo Migliaccio

**Affiliations:** 1 Dipartimento di Medicina di Precisione, Università della Campania ‘L. Vanvitelli’- Via L. De Crecchio, 7-80138 Naples, Italy; 2 Dipartimento Multidisciplinare di Specialità Medico- Chirurgiche e Odontoiatriche, Università della Campania ‘L. Vanvitelli’- Via L. De Crecchio, 6-80138 Naples, Italy

**Keywords:** Diseases, Endocrine system and metabolic diseases

## Abstract

Aging induces a slow and progressive decrease in muscle mass and function, causing sarcopenia. Androgens control muscle trophism and exert important anabolic functions through the binding to the androgen receptor. Therefore, analysis of the androgen receptor-mediated actions in skeletal muscle might provide new hints for a better understanding of sarcopenia pathogenesis. In this study, we report that expression of the androgen receptor in skeletal muscle biopsies from 20 subjects is higher in young, as compared with old subjects. Co-immunoprecipitation experiments reveal that the androgen receptor is complexed with filamin A mainly in young, that in old subjects. Therefore, we have in depth analyzed the role of such complex using C2C12 myoblasts that express a significant amount of the androgen receptor. In these cells, hormone stimulation rapidly triggers the assembly of the androgen receptor/filamin A complex. Such complex prevents the senescence induced by oxidative stress in C2C12 cells, as disruption of the androgen receptor/filamin A complex by Rh-2025u stapled peptide re-establishes the senescent phenotype in C2C12 cells. Simultaneously, androgen stimulation of C2C12 cells rapidly triggers the activation of various signaling effectors, including Rac1, focal adhesion kinase, and mitogen-activated kinases. Androgen receptor blockade by bicalutamide or perturbation of androgen receptor/filamin A complex by Rh-2025u stapled peptide both reverse the hormone activation of signaling effectors. These findings further reinforce the role of the androgen receptor and its extranuclear partners in the rapid hormone signaling that controls the functions of C2C12 cells. Further investigations are needed to promote clinical interventions that might ameliorate muscle cell function as well the clinical outcome of age-related frailty.

## Introduction

The demographic trend towards aging of human populations has attracted the focus of researchers, clinicians and pharmaceutical companies on age-related diseases. Aging and several clinical conditions are often accompanied by muscle wasting. Sarcopenia, a major clinical problem for older people, is defined as an age-associated loss of skeletal muscle mass and function [1]. It was estimated that, after the age of 50, the reduction in muscle cross-sectional area (CSA) is ~1% per year [2, 3] related to a decline in both muscle fiber size and number [4, 5]. An operational definition of sarcopenia combines assessment of muscle mass, muscle strength and physical performance [6, 7].

Sex steroid hormones, mainly estrogens and androgens, are involved in maintaining the homeostasis and strength of skeletal muscle. Androgens act in various nonreproductive cells, including muscle and bone cells, through the androgen receptor (AR) [8–11]. Testosterone, the main androgen in skeletal muscle [12], increases the muscle size and strength in young and older men [13]. It also induces hypertrophy in both type I and type II muscle fibers, with concomitant increase in satellite cell number and myonuclear accretion [14, 15]. In addition, androgens promote the differentiation of mesenchymal multipotent cells into the myogenic lineage, while inhibiting adipogenesis [15]. Satellite cells and myo-nuclei are predominant sites of AR expression in muscle [16], indicating that androgens increase muscle mass mainly by stimulation of satellite cells [17]. Therefore, androgens are considered “anabolic steroids” and promising androgen analogues have been designed and assessed in preclinical and clinical studies, as potential selective androgen receptor modulators (SARMs).

The androgen anabolic effect on muscle becomes evident at puberty, when boys gain 35% more muscle mass than girls. Hypogonadal young men have, indeed, atrophied muscle and androgen supply increases muscle mass and strength. Androgen levels, however, begin to naturally decline with aging in men and this decline is continuous throughout their lifetime. Nevertheless, a number of factors, including obesity, inactivity, trauma, diet, diseases, and drugs might reduce the androgen levels at all ages [9]. Whatever the cause, the androgen decline is often accompanied by sarcopenia. As such, the molecular mechanism of androgen action in skeletal muscle is under intense investigation.

AR is a ligand-regulated nuclear transcription factor that mediates the differentiation and proliferation of target tissues. In addition to regulating gene expression, the androgen-coupled AR rapidly activates several signaling pathways that trigger cell cycle progression, migration and differentiation in various cell types [18]. These effects occur through interaction of AR with effectors and/or scaffolds, such as the tyrosine kinase Src [19] and filamin A (FlnA) [20], or through a cross-talk between AR and other receptors, including epidermal growth factor receptor (EGF-R; [21]), the insulin growth factor receptor I (IGF-RI; [22]) or the sex hormone binding globulin (SHBG) receptor (SHBGR) [23]. Whatever the upstream mechanism, the rapid androgen action increases the intracellular Ca^++^ levels or leads to activation of downstream signaling effectors (i.e., MAPK, Akt, PKA, Rac1, FAK, and paxillin) in various cell types [18, 24]. Notably, Rac1, FAK, and MAPK are involved in muscle development [25, 26] and sarcopenia [27].

The ligand activation of classical AR triggers genomic and nongenomic events leading to anabolic effects in skeletal muscle cells [9, 28]. However, a noncanonical AR, associated with the plasma membrane [29] or localized in *caveolae* [30], has been detected in C2C12 myoblasts. From these compartments, the receptor would exert its actions, including protection from apoptosis. However, sequencing and cloning of this noncanonical receptor still remain pending. It has been also proposed that the AR-mediated increase in skeletal muscle mass is partly mediated by the IGF-I system, which leads to AR phosphorylation, with the consequent nuclear translocation and enhancement of the AR-mediated transcriptional activity [31]. Such cross-talk might provide additional mechanisms to increase muscle size. Despite these findings, the role of androgen/AR axis in skeletal muscle is still under investigation.

The Fln family consists of three homologous proteins, A, B and C that command cell motility. Mutations in Fln genes (A and B) cause, indeed, a wide range of brain, bone, skeleton and heart developmental malformations, likely due to severe defects in embryonic cell migration as well as to the failure of Fln in interacting with other proteins [32]. FlnA and its proteolytic fragments directly interact with AR to regulate nuclear translocation and transcriptional activity of AR [33, 34] as well as androgen-dependence and metastatic phenotype of human prostate cancer [35–37]. We previously reported a role for FlnA in the androgen signaling leading to motility, invasion and differentiation in quite different cell types [20, 38–41]. The findings so far collected indicate, however, that FlnA intersects the androgen action in various cell types at different levels and in different cellular compartments by directly anchoring AR or signaling effectors that contribute to hormonal effects.

In this report, we provide new clues that explain the regulatory role of the androgen-induced AR/FlnA bipartite complex in senescence induced by oxidative stress in skeletal muscle cells. Findings in C2C12 cells and muscle biopsies from young or old healthy subjects suggest that the AR/FlnA complex represents a promising molecular signature of the skeletal muscle’s trophism.

## Results

### Expression of AR negatively correlates with aging in skeletal muscle biopsies. Analysis of AR expression and intracellular localization in C2C12 cells

**Fig. 1:**
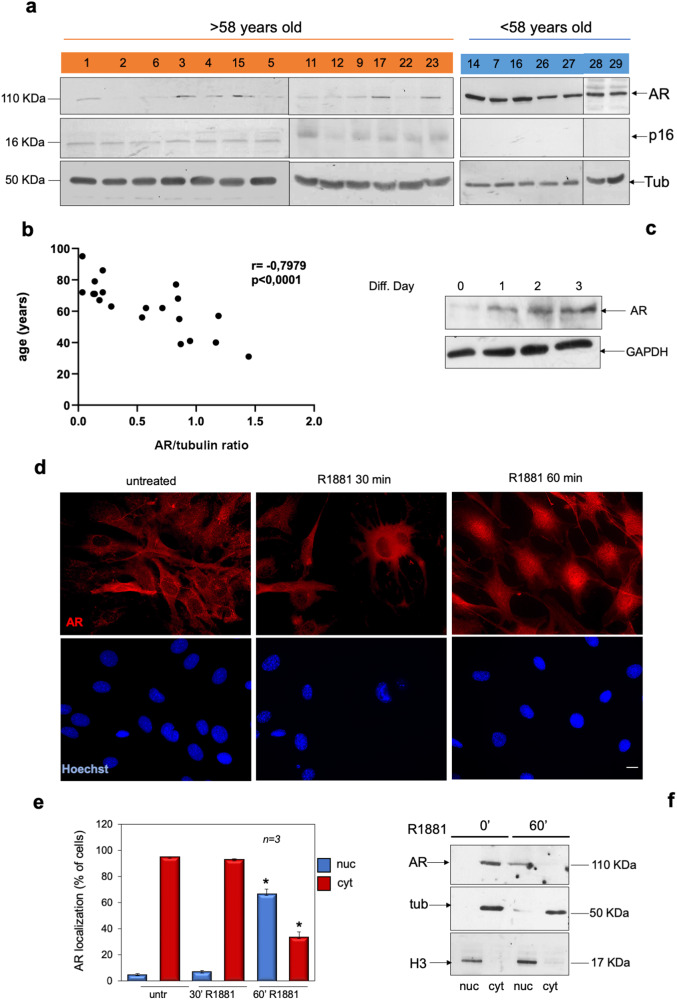
Analysis of AR in human skeletal muscle biopsies and C2C12 cells. **a** Lysate proteins from human skeletal biopsies derived by patients over 58 years (number 1, 2, 6, 3, 4, 15, 5, 11, 12, 9, 17, 22, and 23; in orange) or under 58 years (14, 7, 16, 26, 27, 28, and 29; in blue) of age were prepared and analyzed by WB, using antibodies against the indicated proteins. **b** The correlation between AR protein levels, which were normalized using the corresponding tubulin expression, and the patients age was calculated by Spearman correlation test, using GraphPad. The *r* coefficient and *P* value are reported on the top of the panel. **c** lysates from C2C12 cells were prepared and proteins were analyzed by WB, using antibodies against the indicated proteins. **d** Quiescent C2C12 cells on coverslips were left untreated or treated with 10 nM R1881 for the indicated times (30 and 60 min). Cells were stained for AR (red) or nuclei (Hoechst) and analyzed by IF, as described in “Materials and methods”. Representative images captured from one experiment in **d** are shown. Bar, 5 µm. **e** AR localization was expressed as percentage of cells showing a predominant cytoplasmic (cyt) or nuclear (nuc) AR localization. Means and SDs are shown; *n* represents the number of experiments. *P < 0.05 for the indicated experimental points versus the corresponding untreated control cells. **f** Quiescent C2C12 cells were left untreated (0’) or treated with 10 nM R1881 for 60 min. Cytoplasmic- (cyt) or nuclear- (nuc) enriched fractions were prepared as described in “Materials and methods” and proteins were analyzed by western blot, using the anti-AR antibody. The fractions were also analyzed for expression of tubulin and histone H3, as cytoplasmic and nuclear marker, respectively.

Skeletal muscle biopsies positive for myoD expression (Supplementary Fig. 1Sa) were obtained from 20 healthy subjects and split into two groups, old (>58 years; mean age 72.6) and young (< 58 years; mean age 46.5). The WB analysis in Fig. 1a shows that lysate proteins derived from young subject’s (ys) biopsies express higher levels of AR, as compared with older subjects (os). Using the same lysate samples, we then analyzed the expression of the cyclin-dependent kinase inhibitor (CDKI) p16, a regulator of the senescence program [42]. The WB analysis reveals higher expression of p16 in os, as compared with ys subjects (Fig. 1a). Spearman’s rank correlation shows a significant negative correlation between AR expression, which was normalized using tubulin levels, and age (*r* = −0.7979; *P* < 0.0001; Fig. 1b).

To further investigate the role of AR in skeletal muscle, we then used C2C12 myoblasts [43]. We firstly analyzed the AR expression in C2C12 myoblasts by WB technique. Figure 1c shows that the receptor expression levels increase in C2C12 cells over the days of serum depletion. We next analyzed by immunofluorescence (IF) analysis the intracellular AR localization in C2C12 cells untreated or treated with physiological (10 nM) concentration of the synthetic androgen, R1881. Images in Fig. 1d show that AR is prevalently localized in the cytoplasm of untreated C2C12 cells. Androgen stimulation induces within 60 min the nuclear translocation of AR. Data derived from several independent experiments are presented in Fig. 1e. The absence of fluorescence in C2C12 cells stained with the secondary antibody alone indicates the specificity of IF approach (Supplementary Fig. 1Sb). To confirm the data observed by IF microscopy, we also analyzed the AR subcellular localization by WB of cytoplasmic- and nuclear-enriched fractions from C2C12 cells. Figure 1f shows that androgen treatment increases over the time the bulk of AR levels in nuclear-enriched fractions of C2C12 cells, further indicating that these cells express classical AR. Cytoplasmic and nuclear fractions were also probed for α-tubulin and histone H3, as cytoplasmic and nuclear marker, respectively.

### Androgens protect C2C12 cells from senescence induced by H_2_O_2_

**Fig. 2:**
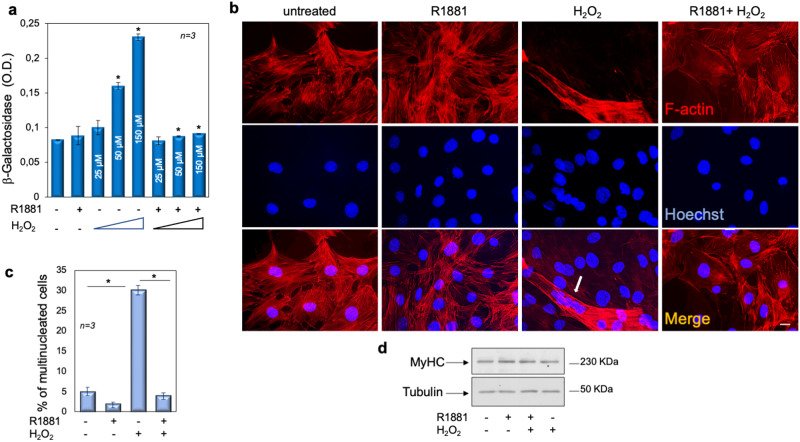
Androgens protect C2C12 cells from senescence induced by sub-lethal concentrations of H_2_O_2_. **a**–**d** C2C12 cells were left untreated or pre-treated with 10 nM R1881 for 4 h. **a** Cells were challenged with different concentrations (25, 50, and 150 µM) of H_2_O_2_ for 36 h. β-gal absorbance was detected by evaluating the absorption at 420 nm of o-nitrophenol. **b** Cells on coverslips were challenged with 150 µM H_2_O_2_, stained and analyzed for F-actin (red) and nuclei with Hoechst (blue). The arrow indicates multinucleated cells. bar, 5 µm. **c** Multinucleated cells were counted and represented in the graph. **d** shows the WB of lysates proteins from cells challenged as (**b**), using the antibodies against the indicated proteins. **a**, **c** Means and SDs are shown; *n* represents the number of experiments. The asterisk (*) indicates *P* < 0.05.

A role for AR has been proposed in many age-related diseases [44] and the androgen/AR axis mediates, through nongenomic mechanism, the cellular senescence in prostate cancer cells [45]. Androgens naturally decrease with age and the loss of testosterone is associated with the age-related decline in muscle mass. Nevertheless, a role for AR in muscle senescence is still not well established. Therefore, we investigated the effect of androgen treatment on senescent phenotype of C2C12 cells. Exposure to sub-lethal concentrations of H_2_O_2_ induces senescence in various cell types [46]_._ Therefore, untreated or androgen-treated cells were exposed to a range (25–50–150 µM) of H_2_O_2_ concentration and cellular senescence was assessed by detecting the absorbance at 420 nm of o-nitrophenol, after 36 h (Fig. 2a). Exposure to 50 and 150 µM H_2_O_2_ results in induction of β-gal positive cells, while androgen pre-treatment reduces this effect. Thus, we used this experimental setting (150 μM H_2_O_2_ for 36 h) throughout the subsequent studies. Since no single trait might solely define the cellular senescence, we confirmed this phenotype by evaluating in vitro the multinucleation as the additional hallmark of senescent cells [47]. Figure 2b and quantification of data in panel c show that H_2_O_2_ increases by ∼sixfold the multinucleated cell fraction. Pre-treatment of cells with androgens prevents such an effect. To exclude the contribution of cell differentiation in the reported effects, we also analyzed the myosin heavy chain (MyHC) expression [48]. Regardless of experimental conditions, the WB in Fig. 2d shows that the MyHC content does not significantly change, indicating that the observed increase in cellular multinucleation is not due to cell differentiation.

**Fig. 3:**
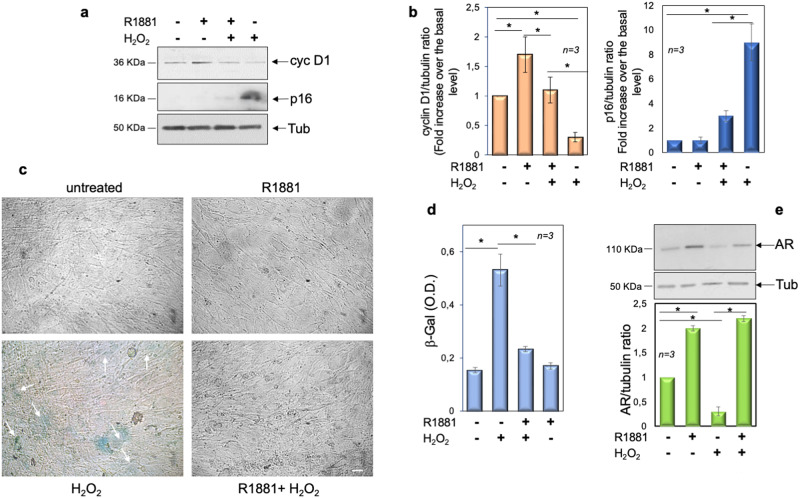
Androgens protect C2C12 cells from senescence induced by H_2_O_2_. **a**–**e** C2C12 cells were untreated or pre-treated with 10 nM R1881. After 4 h, cells were treated with 150 µM H_2_O_2_ for 36 h. **a** The WB of lysates proteins from C2C12 cells, using the antibodies against the indicated proteins are shown. Images are representative of three different experiments. Expression levels of proteins were analyzed by densitometry analysis, using NIH Image J Software. The ratio between cyclin D1/Tubulin (**b**, left panel) and p16/Tubulin (**b**, right panel) was evaluated. **c** Cells were processed for β-galactosidase detection through cytochemistry at pH 6, as described in “Materials and methods” and pictures were acquired. The arrows indicate the blue, senescent cells; bar, 5 µm. **d** β-gal absorbance was detected by evaluating the absorption at 420 nm of o-nitrophenol. **e** WB of lysates from C2C12 cells using the antibodies against the indicated proteins are shown. The WB in the upper panel are representative of three different experiments. Expression levels of AR were analyzed by densitometry analysis, using NIH Image J Software. The ratio between AR/Tubulin was evaluated. **b**, **d**, **e** Means and SDs are shown; *n* represents the number of experiments. The asterisks (*) indicate *P* < 0.05.

Cell cycle arrest by the onco-suppressor network, mainly p16, p21, pRb, and p53, is a hallmark of senescence [49]. The WB analysis in Fig. 3a, together with quantification of the data in panel b, show that H_2_O_2_ promotes upregulation of p16 and downregulation of cyclin D1. H_2_O_2_ leaves unaffected p21 or p53 (not shown). Androgens slightly, but significantly increase the cyclin D1 expression levels, attenuate the effect of H_2_O_2_ on cyclin D1 expression, and prevent the upregulation of p16 induced by H_2_O_2_. Analysis of senescent cells by contrast-phase microscopy consistently reveals that H_2_O_2_ results in the accumulation of a distinctive blue color in a significant number of cells, given the activity of senescence-associated β-galactosidase (SA-β-gal), which catalyzes the hydrolysis of X-gal. Hormone treatment completely prevents this effect (Fig. 3c). Data were confirmed by analysis of β-gal absorbance in several independent experiments (Fig. 3d). Simultaneously, we observed that androgens stabilize the bulk of AR protein levels, as compared with the WB analysis of the receptor from untreated cells (upper section in panel e). H_2_O_2_ treatment significantly reduces the AR expression, while the addition of hormone protects the receptor from degradation (upper section in panel e). Quantification of images from WB analysis is shown in the lower section (panel e). These data are consistent with the findings that AR might undergo degradation [50] and that hormone treatment stabilizes and protects the receptor over time [51]. Taken together, our results suggest that oxidative stress, typical of age-related disorders, such as sarcopenia and frailty, might be characterized by the loss of AR in skeletal muscle cells. Androgen supply would restore the receptor level, thus preventing skeletal muscle cell senescence. Consistent with our hypothesis, a reduction of muscle AR levels has been detected during age-related muscle loss [52] and upregulation of AR by androgens has been reported in rat and human skeletal muscle [53–55].

### The AR/FlnA complex in human skeletal muscle biopsies and C2C12 cells

The actin-binding and actin-crosslinking proteins, Flns A, B, and C contain a N-terminal actin-binding domain followed by 24 immunoglobulin-(Ig-) like domains, the last of which is responsible for dimerization of Flns. These proteins recognize a *plaethora* of partners, such as AR, intracellular signaling molecules, ion channels, transcription factors, cytoskeletal and cell adhesion proteins. Consistently, a lot of functions are attributed to Flns, including the organization and stabilization of the actin cytoskeleton, its anchorage to transmembrane proteins at sites of cell-substrate- and cell-cell-adhesion, the integrity of heart, brain, and skeletal muscle [32].

**Fig. 4:**
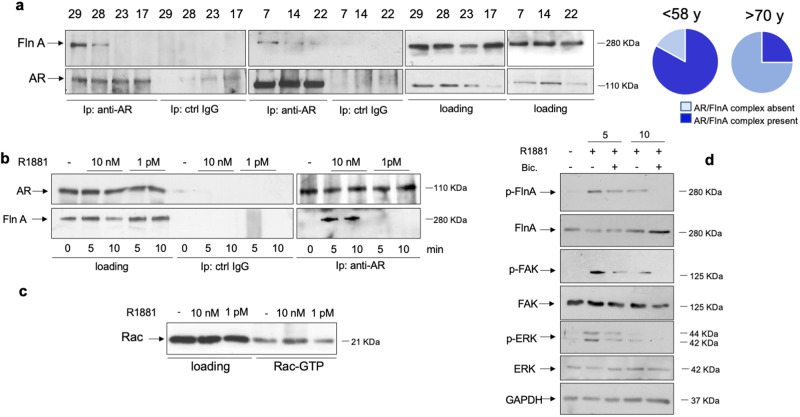
Analysis of AR/FlnA complex in skeletal muscle biopsies and C2C12 cells. The signaling effectors activated by androgens/AR axis in C2C12 cells. **a** Lysate proteins from human skeletal biopsies derived by patients under 58 (number 29, 28, 7, and 14) or over 70 years (23, 17, and 22) of age were prepared and immune-precipitated using the anti-AR antibody (anti-AR) or control IgG (ctrl IgG). Proteins in immune complex were detected by WB, using antibodies against the indicated proteins. The right sections in (**a)** show the WB of lysate proteins using the antibodies against the indicated proteins (loading). Pie charts show that 75% and 25% of muscle biopsies from patients under 58 or 70 years, respectively, exhibit the AR/FlnA complex. **b**, **c** C2C12 cells were made quiescent and then left unchallenged or challenged for 5 or 10 min with 10 nM or 1 pM R1881. **b** The left section shows the WB of lysate proteins (loading) with antibodies against the indicated proteins. Similar amounts of lysate proteins were immune-precipitated using the anti-AR antibody (right section, anti-AR) or control IgG (middle section, ctrl IgG). Proteins in immune complex were detected by WB, using the antibodies against the indicated proteins. **c** lysate proteins were used for Rac pull-down assay. The WB with anti-Rac antibody revealed the eluted, active Rac (Rac-GTP, right section). The total amount of Rac expressed in the corresponding lysates was also detected (left section). **d** cells were left unchallenged or challenged for 5 or 10 min with 10 nM R1881, in the absence or presence of 10 μM bicalutamide (bic). Lysate proteins were analyzed for FlnA, FAK and ERK phosphorylation using the anti-p-FlnA Ser 2152, or anti-p-Tyr397FAK, or anti-p-Tyr204 ERK antibodies, respectively. The filters were stripped and re-probed using anti-FlnA, -FAK, and -ERK antibodies. The levels of GAPDH were also detected as loading control, using appropriate antibodies.

We recently showed that the androgen-induced AR/FlnA complex assembly and its downstream pathway is involved in cell motility, invasion and differentiation of target cells [20, 36–41]. Given these findings, we hypothesized a role for FlnA in the biological responses activated by androgens in skeletal muscle. Therefore, we first tracked the AR/FlnA complex in human skeletal muscle biopsies from healthy subjects at different age. As indicated in “Materials and methods”, only lysate proteins expressing significant amounts of both AR and FlnA were collected from young (<58 years) or old (>70 years) subjects and prepared. Lysate proteins containing similar amounts of AR and FlnA were used in Co-IP experiments. Notably, the AR/FlnA complex was mainly detected in young subjects (left section in Fig. 4a). These findings are represented as a pie chart in Fig. 4a (right section). Although such an experimental approach does not allow any speculation about the androgen effect, our findings in human skeletal muscle biopsies corroborate the hypothesis that AR/FlnA complex protects the skeletal muscle cells from aging.

We next analyzed the AR/FlnA complex assembly in C2C12 cells. The cells were challenged with two different androgen concentrations, 10 nM or 1 pM, since the maximal complexation between AR and FlnA occurs at optimal (10 nM) androgen concentration [20]. Similar amounts of AR or FlnA were detected by WB of lysate proteins. Ten nM R1881 stimulation of C2C12 cells induces within 5 min the Co-IP of AR with FlnA. By contrast, no Co-IP is detectable in cells challenged with 1 pM R1881. The absence of AR or FlnA in lysates immunoprecipitated with the control antibody (ctrl IgG) indicates the specificity of our approach (Fig. 4b).

We next analyzed the hormone effect on activation of Rac, one of the most important drivers of FlnA signaling [56]. Ten nM R1881 strongly enhances the amount of activated Rac (Rac-GTP) in pull-down assay, while no effect is detected at 1 pM R1881 (panel c). Simultaneously, 10 nM R1881 stimulates within 5 min the phosphorylation of FlnA at serine 2152 (Supplementary Fig. 1Sc and Fig. 4d). Such modification is required for FlnA to induce cell shape changes and membrane ruffling [57]. At last, 10 nM R1881 treatment triggers within the same time frame the FAK tyrosine 397 phosphorylation as well ERK activation. The anti-androgen bicalutamide reverts the androgen effects, indicating the involvement of classic AR in these rapid effects (panel d). This set of experiments strongly suggests that the androgen-triggered AR/FlnA complex assembly leads to activation of the downstream pathways controlling motility, adhesion and contractility of cells, including muscle cells.

### The AR/FlnA complex protects skeletal muscle C2C12 cells from senescence

**Fig. 5:**
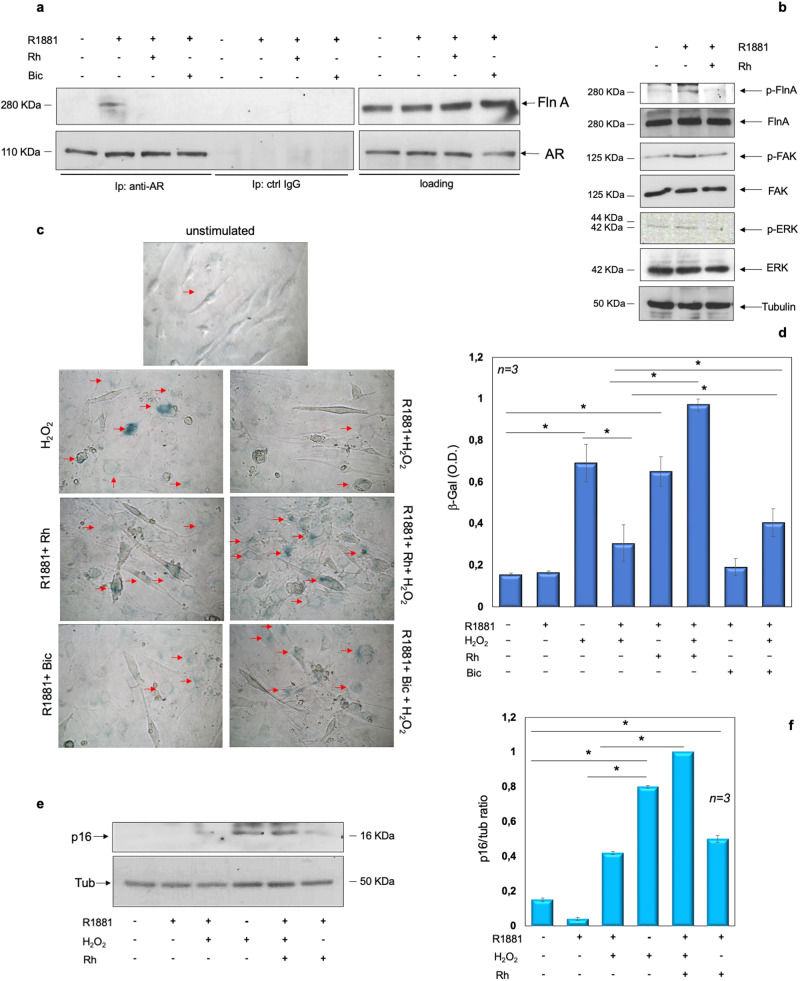
The role of the androgen-triggered AR/FlnA complex assembly in signaling activation and senescence of C2C12 cells. **a**–**f** quiescent C2C12 cells were used. **a** cells were left unchallenged or challenged for 5 min with 10 nM R1881, in the absence or presence of either 100 nM Rh-2025u or 10 µM bicalutamide (Bic). Lysate proteins were analyzed by WB, using the antibodies against the indicated proteins (loading control in the right section). Similar amounts of lysate proteins were immune-precipitated using the anti-AR (left section, anti-AR) or control (middle section, ctrl IgG) antibodies. Proteins in immune complex were detected by WB, using antibodies against the indicated proteins. **b** cells were unchallenged or challenged for 5 min with 10 nM R1881, in the absence or presence of 100 nM Rh-2025u. Lysate proteins were analyzed for FlnA, FAK and ERK phosphorylation using the anti-p-FlnA Ser 2152, or anti-p-Tyr397FAK, or anti-p-Tyr204 ERK antibodies. The filter was stripped and re-probed using anti-FlnA, -FAK, and -ERK antibodies. Tubulin levels were revealed as loading control, using appropriate antibodies. **c**–**f** Cells were untreated or pre-treated with 10 nM R1881. Four hours later, the cells were challenged with 150 µM H2O2 for 36 h. **c** β-galactosidase was detected by cytochemistry at pH 6 and images were acquired. The arrows indicate the blue, senescent cells. Bar, 5 µm. **d** β-gal absorbance was detected by evaluating the absorption at 420 nm of o-nitrophenol. Shown in (**e**) are the WB of lysate proteins from C2C12 cells, using antibodies against the indicated proteins. They are representative of three different experiments. Expression levels of proteins were analyzed by densitometry analysis, using NIH Image J Software. The ratio between p16/Tubulin (**f**) was evaluated. **d**, **f** The asterisk (*) indicates *P* < 0.05.

In search for a link between AR/FlnA complex and C2C12 cell senescence, we then used the stapled Rh-2025u peptide (Supplementary Fig. 1Sd) that specifically perturbs the androgen-induced AR/FlnA complexation [38–41]. Challenging the cells with 10 nM R1881 stimulates within 5 min the association of AR with FlnA in C2C12 cells. The addition of Rh-2025u peptide or bicalutamide reverses this association. The absence of AR and FlnA in cell lysates immune-precipitated with control antibodies confirms the specificity of Co-IP approach (Fig. 5a). Similar amounts of AR or FlnA were detectable in cell lysates, regardless of experimental conditions (right panels in Fig. 5a). Simultaneously, 10 nM R1881 stimulates within 5 min the phosphorylation of FlnA at serine 2152, as well as FAK and ERK activation. The Rh-2025u peptide reverts the androgen effects (panel b), indicating that the androgen-triggered AR/FlnA complex controls activation and phosphorylation of the downstream signaling components involved in motility, adhesion, and contractility of cells, including muscle cells.

In the same experimental setting, C2C12 cell treatment with H_2_O_2_ induces cell senescence, as shown by contrast-phase microscopy. Ten nM R1881 reverses such phenotype, which is re-established by the addition of Rh-2025u peptide. Albeit at different extent, bicalutamide gives similar results. Irrespective of H_2_O_2_ treatment, Rh-2025u peptide even induces senescence in androgen-treated C2C12 cells (Fig. 5c) and similar data were observed by measuring the β-gal absorbance in several independent experiments (Fig. 5d). Analysis of p16 expression by WB technique gives superimposable results (Fig. 5e, f).

Taken together, these findings suggest that specific perturbation of AR/FlnA complex assembly impairs the androgen effect and commits the cells towards senescence. Overall, our data suggest that FlnA plays a permissive role in muscle cell trophism and cooperates with AR in sustaining the androgen action in skeletal muscle cells.

## Discussion

Findings from clinical and preclinical studies support each other the anabolic role of androgens in skeletal muscle. This effect occurs through several intracellular targets and biochemical pathways, although their identification under physiological and clinical conditions still remains a challenge.

In this study, we have analyzed the role of rapid, nongenomic androgen action in skeletal muscle senescence. Experiments in C2C12 myoblasts indicate that androgens prevent the senescence induced by H_2_O_2_ in these cells. The hormonal effect occurs through the AR/FlnA complex assembly, as perturbation of the complex assembly by Rh-2025u stapled peptide completely re-establishes the senescent phenotype in C2C12 cells, even in the absence of oxidative stress. The latter finding points to the critical role of AR/FlnA complex in muscle cell functions. Analysis of the complex assembly reveals that interaction of the receptor with FlnA occurs within 10 min upon stimulation of cells with optimal androgen concentration (10 nM), as shown by Co-Ip experiments in C2C12 myoblasts. By contrast, no effect can be observed at low hormone concentration (1 pM). Similar findings were reported using quite different nonreproductive cells, such as fibroblasts, cancer-associated fibroblasts, fibrosarcoma and neuronal cells [20, 40, 58]. The correlation between optimal hormone concentration and AR/FlnA complexation likely reflects the loss of beneficial hormonal effects in the skeletal muscle. Aging men, indeed, frequently experience the negative effects of a decrease in circulating testosterone, resulting in a clinical syndrome (also known as “androgen-deficiency”) characterized by an increased risk of metabolic, vascular and neurodegenerative diseases as well as the age-related decline in muscle mass [59]. The findings we observed in young and old healthy subjects support the role of AR/FlnA complex in skeletal muscle well-being.

Once assembled, the AR/FlnA complex triggers the rapid activation of various downstream components, including Rac, FAK and MAPKs. Activation of these effectors by the androgen-induced AR/FlnA complex is not unexpected. Nevertheless, the present results in muscle cells are completely novel. Additionally, they are consistent with the role of Rac in muscle development [25] as well as the involvement of FAK and MAPK activation in muscle atrophy associated with cachectic or sarcopenic conditions [26, 27]. The finding that Rh-2025u peptide perturbs the androgen-induced activation of these effectors further indicates that the AR/FlnA complex controls signaling circuits involved in muscle trophism and performance. At last, the results obtained using the AR blocker bicalutamide strongly support the hypothesis that ligand stimulation of a classic AR, poised at cytoplasm or near the cell membrane, is responsible for the reported effects. However, other mechanisms (i.e, cross-talk with growth factor, vitamin D or cytokine’s signaling pathways) or involvement of G protein-coupled receptors (GPCRs), which bind and are activated by androgens, cannot be excluded. Findings obtained using bicalutamide are consistent with the clinical observation that while androgen deprivation therapy delays prostate cancer (PC) progression and alleviates cancer-related symptoms in some PC patients, it is also associated with unwanted effects that increase the risk of sarcopenia, frailty and co-morbidities (e.g, type II diabetes and cardiovascular diseases [60–65]).

The findings concerning AR/FlnA complexation in C2C12 cells and human muscle specimens call for additional comments. Previous reviews have highlighted the role of Fln family members, A, B and C in cell functions [32, 66–68]. Among the three members, FlnC seems to play a role in muscle cell function, as it is predominantly expressed in skeletal muscles and its loss in mice causes a severe muscle phenotype [69]. Nevertheless, a role for FlnA in muscle development has been reported in a mouse model of FlnA deficiency [70]. Consistent with its broad expression, FlnA might, hence, contribute to several developmental processes and tissue functions. These findings, together with the observation that FlnA binds a *plaethora* of signaling components and heterodimerizes with FlnB or C [67], expand the combination of interactions that might affect specific tissue functions. Thus, the possibility that combination of AR with Fln heterodimers (i.e., FlnA/FlnB or FlnA/FlnC) regulates the muscle cell functions cannot be excluded, despite our previous findings concerning the interaction of AR with FlnA, but not FlnB or FlnC [20]. Lastly, a role for FlnA in cell senescence has been exploited in different experimental conditions. Inhibition of FlnA interaction with Drp1, a modulator of mitochondrial dynamics, attenuates cardiomyocyte senescence after myocardial infarction [71] and involvement of FlnA in thrombotic or bleeding disorders associated with aging has been previously reported [72]. A nonsense mutation in the Fln gene, *fln-2* (a distant ortholog of FlnA in humans) reduces early mortality caused by pharyngeal infection in *C. elegans* [73]. By restoring the native conformation of FlnA, the small molecule PTI-125 improves the synaptic plasticity in Alzheimer’s disease mouse model [74]. Again, genetic variations and protein abundance of FlnA induce tau aggregation and tau pathologies causing neurodegeneration and progressive supranuclear palsy [75]. These and other reports support a role for FlnA in senescence and aging. Of note, the androgen circuit seems involved in the aforementioned diseases, as hormone decline is associated with a wide range of cardiovascular, metabolic and neurological disorders [18].

In conclusion, this study further expands the knowledge of the androgen-triggered AR/FlnA complex we previously described in mesenchymal cells. Future investigations might provide new hints in the identification of new targets and therapeutics in skeletal muscle diseases.

## Materials and methods

### Chemicals and cell cultures

Unless otherwise stated, R1881 and bicalutamide (Sigma-Aldrich, St. Louis, MO, USA) were used at 10 nM and 10 μM, respectively. The stapled peptide Rh-2025u was synthesized [38] and used at 100 nM (final concentration). Mouse myoblast C2C12 cell line was from ATCC. Cells were maintained at 37 °C in humidified 5% CO_2_ atmosphere. Media and supplements were from Gibco (Thermo-Fisher, Waltham, MA, USA). C2C12 cells were cultured in DMEM supplemented with 10% fetal bovine serum (FBS), streptomycin (100 U/ml), glutamine (2 mM). Three days before stimulation, C2C12 cells were made quiescent using phenol red-free DMEM medium containing 1% charcoal-stripped serum (CSS), streptomycin (100 U/ml), and glutamine (2 mM). Cells were routinely monitored for Mycoplasma contamination.

### Patient details, skeletal muscle biopsy, and tissue lysate preparation

Twenty patients were enrolled at the Orthopedics Unit at University Hospital “L. Vanvitelli”. Patients who underwent a primary total hip arthroplasthy (THA) and/or a primary total knee arthroplasty (TKA) were considered eligible. In patients undergoing THA, the muscle biopsy was collected using a modified version of the Herdinge-Baur direct lateral approach. Specifically, the anterior third of the gluteus medius was detached from the greater trochanter, and biopsies were collected from detached fibers of the gluteus medius. In patients undergoing TKA, a median parapatellar approach was done, and detached fibers from Vastus medialis were collected.

Table 1 contains the numbered list of patients, together with their corresponding ages. Of note, the list of patients includes 7 young (age <58 years) and 13 old (age >58 years) subjects, as young subjects are less fragile and less exposed to spontaneous traumas. Hence, they do not frequently undergo surgical interventions. A written informed consent was signed by each patient and collected. This study was approved by the Ethics Committee of the University of Campania “L. Vanvitelli”, Napoli, Italy (protocol number 0017043/i/2023). Fresh skeletal muscle tissues were immediately frozen at −80 °C and processed within 1–2 weeks. Before the lysis, tissue samples were cut into cubes of ~1 mm using a razor blade on a glass plate in ice. Skeletal muscle tissues were kept on ice in 1 ml of RIPA buffer (20 mM Tris-HCL pH 7.4, 150 mM NaCl, 1 mM EDTA, 1% Triton X-100, 1% sodium deoxycholate, 0.1% SDS, containing 1 mM PMSF and 5 µg/ml aprotinin and leupeptin). Tissue homogenization was done in ice, using a Dounce homogenizer (Bellco Glass, Vineland, NJ, USA) with 10x looser fitting pestle A, followed by 10× looser fitting pestle B. Tissue lysates were centrifuged at 13,000 rpm for 3 min at 4 °C and supernatants were collected for protein assay, using the Bio-Rad Protein Assay Dye Reagent Concentrate (Bio-Rad Laboratories, Hercules, CA, USA).

**Table 1:** Numbered list of patients and their corresponding age.

Patient’s number	age
1	62
2	63
3	68
4	77
5	86
6	72
7	55
9	95
11	67
12	72
14	57
15	62
16	56
17	79
22	71
23	71
26	40
27	29
28	41
29	39

### Cytoplasmic and nuclear fractionation

Cells were collected with a scraper, washed with PBS, suspended in cold Harvest buffer (10 mM HEPES, pH 7.9, 50 mM NaCl, 0.5 M sucrose, 0.1 mM EDTA, 0.5% Triton X-100) containing 1 mM sodium orthovanadate, 1 mM DTT, 100 mM NaF, 17.5 mM β-glycerophosphate, 1 mM PMSF, 4 μg/ml Aprotinin as well as protease inhibitor cocktail (LAP), then incubated on ice for 5 min. Homogenates were centrifuged at 2500 rpm to collect nuclei. The supernatants were clarified at 15,000 rpm for 15 min and cytosolic extract was used. For nuclear extraction, nuclei were washed twice with washing buffer (10 mM HEPES pH 7.9 containing 10 mM KCl, 0.1 mM EDTA, 0.1 mM EGTA), plus freshly added protease inhibitors (1 mM DTT, 1 mM PMSF, 4 μg/ml Aprotinin and LAP), then incubated with nuclear lysis buffer (10 mM HEPES pH 7.9, 500 mM NaCl, 0.1 mM EDTA, 10 mM EGTA, 0,1% Nonidet P-40), containing 1 mM DTT as well as freshly added protease inhibitors (1 mM PMSF, 4 μg/ml Aprotinin and LAP). The nuclear extract was vortexed at 4 °C for 15 min, then centrifuged at 15,000 rpm for 10 min and used.

### Cell lysates, immune-precipitation (IP), co-immune-precipitation (Co-IP), Rac pull-down assay, and western blot (WB)

Unless otherwise stated, cell lysis was done as reported [76], using 2 mg/ml of protein lysates. Three mg of lysate proteins were used in Co-IP analysis from old-subject skeletal muscle biopsies. One mg of lysate proteins was instead used in Co-IP analysis from young-subject skeletal muscle biopsies. The rabbit monoclonal anti-AR (E354N; Cell Signaling, Beverly, MA, USA) antibody was used to immune-precipitate and detect AR in Co-IP experiments. Rac pull-down assay was done [38], using the Rac activation kit (Millipore, Burlington, MA, USA). For WB analysis, the following antibodies were used: the mouse monoclonal anti-tubulin (E-AB-20036 Elabscience, Houston, TX, USA) or GAPDH (E-AB-48017, Elabscience), the rabbit monoclonal anti-AR (E354N; Cell Signaling), the rabbit polyclonal anti-histone H3 (06-755; Merck-Millipore), anti-myosin heavy chain (MYH1 A6935; ABclonal), anti-phospho-FlnA (Ser 2152; sc-4761S; Cell Signaling) or anti-FlnA (sc-4762S; Cell Signaling) antibodies, the rabbit monoclonal anti-MyoD (MyoD1 antibody [HL1372]; GTX636812; GeneTex, Irvine, CA, USA). P-Tyr118 paxillin was detected using the rabbit monoclonal anti-P-Y118 paxillin antibody (2541, Cell Signaling). Mouse monoclonal anti-paxillin antibody (clone 349; BD Biosciences, San Jose, CA USA) was used to detect total paxillin. Mouse monoclonal anti-phospho-FAK (Y397) (611807; BD Bioscience) or anti-FAK (610088; BD Bioscience) antibodies were used to detect P-Tyr397-FAK or total FAK. Erk and P-Tyr204 Erk were revealed using mouse monoclonal anti-ERK-2 (D2; sc-1647; Santa Cruz Biotecnology, Dallas, Texas, USA) or anti-phospho-ERK (E-4; sc-7383; Santa Cruz Biotecnology) antibodies. The mouse monoclonal anti-p15 INK4B/p16 INK4A (sc-377412) antobody was from Santa Cruz Biotecnology. The ECL system (GE Healthcare) was used to reveal immune-reactive proteins. The full-length uncropped original western blots are uploaded as a single Supplementary File.

### Cytoskeleton analysis, AR staining, and immunofluorescence (IF)

Quiescent C2C12 cells on coverslips were unchallenged or challenged with R1881, in the absence or presence of H_2_O_2_. For cytoskeleton analysis, cells were fixed using diluted (3% wt/vol in PBS) paraformaldehyde (Millipore) and stained with diluted (1:250 in PBS) Texas red-labeled phalloidin (Sigma-Aldrich), as reported [40]. For AR staining, cells on coverslips were fixed and permeabilized [40]. Endogenous AR was visualized using diluted (1:25 in PBS) rabbit monoclonal antibody against the N-terminal domain of AR (clone 105225; Abcam, Cambridge, UK). Diluted (1:300 in PBS) anti-rabbit Texas red-conjugated antibody (Jackson Laboratories, Bar Harbor, Maine, USA) was used as a secondary reagent. Nuclei were stained with Hoechst 33342 (Sigma-Aldrich) and coverslips were inverted and mounted in Mowiol (Sigma-Aldrich). Cells on coverslips were analyzed using a DMLB (Leica, Wetzlar, Germany) fluorescent microscope, equipped with HCX PL Apo ×63 oil and HCX PL Fluotar ×100 oil objectives. Images were captured using DFC365 camera (Leica) and acquired using Leica Suite software. They are representative of at least three experiments, each done in duplicate.

### Detection of senescence through β-galactosidase (β-gal) staining and absorbance

C2C12 cells were unchallenged or challenged with R1881, in the absence or presence of H_2_O_2_. When indicated, the Rh-2025u peptide and bicalutamide were added. For β-gal staining (KTA3030, Abbkine, Wuhan, China), cells were washed twice in PBS, fixed with an appropriate fixation buffer for 15 min at room temperature and washed three times in PBS. Freshly prepared staining solution, containing X-Gal (2 ml/well of six-well plate) was added and cells were incubated at 37 °C in a humidified chamber overnight. The day after, cells were repeatedly washed in PBS. β-galactosidase-expressing cells turned blue and were scored from different fields by contrast-phase microscopy. Representative images from at least three experiments, each done in duplicate, were captured using DFC450 camera (Leica) and acquired using Leica Suite software. For β-gal absorbance detection, the cells were washed in PBS and gently scraped with lysis buffer. They were collected in 1.5-ml tubes, transferred to an ice bucket and centrifuged at 1400 rpm for 2 min at 4 °C. β-gal activity was assayed using a buffer containing NaH_2_PO4 (0.2 M), Na_2_HPO4 (0.2 M), MgCl_2_ (4 M), β-mercaptoethanol (100 mM), O-Nitrophenyl β-D-galactopyranoside (O-NPG, substrate of β-galactosidase; Sigma-Aldrich). It was added to 96-well plate, together with cell lysate. 96-well plates were obscured and left in agitation for 60 min at room temperature. The hydrolysis of colorless O-NPG was measured by the absorption at 420 nm of o-nitrophenol, which has a sensitive yellow color, using the EnSpire apparatus (Perkin-Elmer, MA, USA).

### Statistical analysis

Experiments were done in triplicate and data are presented as mean ± standard deviation. All the comparisons were performed using the paired two-tailed Student’s *t* test on the GraphPad Prism 5.0 software. *P* values < 0.05 were considered statistically significant.

### Supplementary information

Supplementary Information file: Supplementary figure and legend

Uncropped Western blots

## Data Availability

The data generated during the current study are available from the corresponding author on reasonable request. The authors declare that the full-length uncropped original western blots are published in supplemental materials. Other information are available upon request.
