# Supplementary material for: Filamin A cooperates with the androgen receptor in preventing skeletal muscle senescence
Source: Cell Death Discov. 2023 Dec 2;9:437. doi: 10.1038/s41420-023-01737-y (PMC10692324; doi:10.1038/s41420-023-01737-y)
Supplement: Supplementary file 1 — Supplementary Information file: Supplementary figure and legend [file 41420_2023_1737_MOESM1_ESM.pdf]

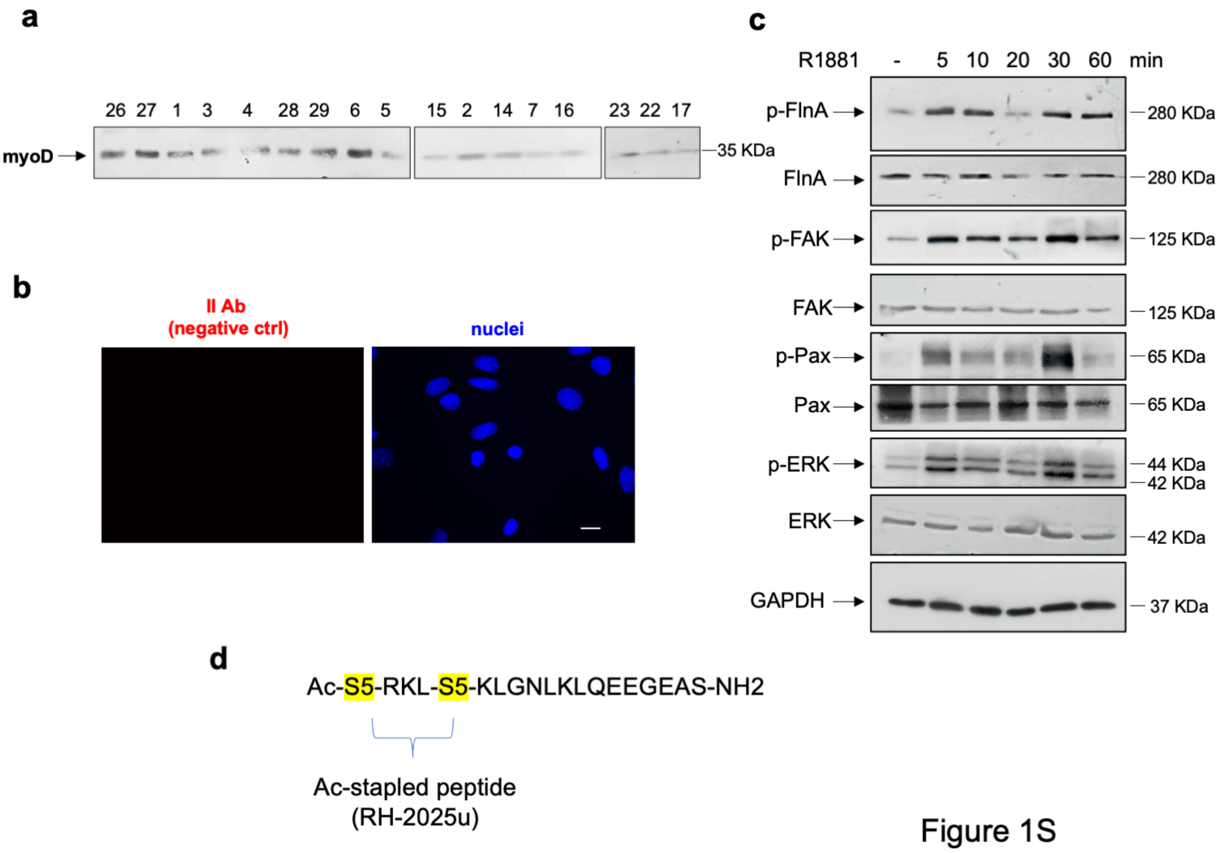

Figure 1S

Figure 1S. In **a)** lysate proteins from human skeletal biopsies were analyzed by WB, using antibodies against the indicated protein. In **b)** quiescent C2C12 cells on coverslips were left untreated and stained with the secondary antibody (red) alone, as negative control. Nuclei were stained with Hoechst and coverslips were mounted using Mowiol and analyzed by IF. Bar, 5µm. In **c)** quiescent C2C12 cells were unstimulated or stimulated for the indicated times with 10 nM R1881. Lysate proteins were analyzed by WB, using antibodies against the indicated proteins. Shown in **d)** is a schematic representation of the modified, stapled Rh-2025u peptide.
