# Supplementary material for: Filamin A cooperates with the androgen receptor in preventing skeletal muscle senescence
Source: Cell Death Discov. 2023 Dec 2;9:437. doi: 10.1038/s41420-023-01737-y (PMC10692324; doi:10.1038/s41420-023-01737-y)
Supplement: Supplementary file 2 — Uncropped Western blots [file 41420_2023_1737_MOESM2_ESM.pdf]

full length uncropped original western blots

Figure 1 a

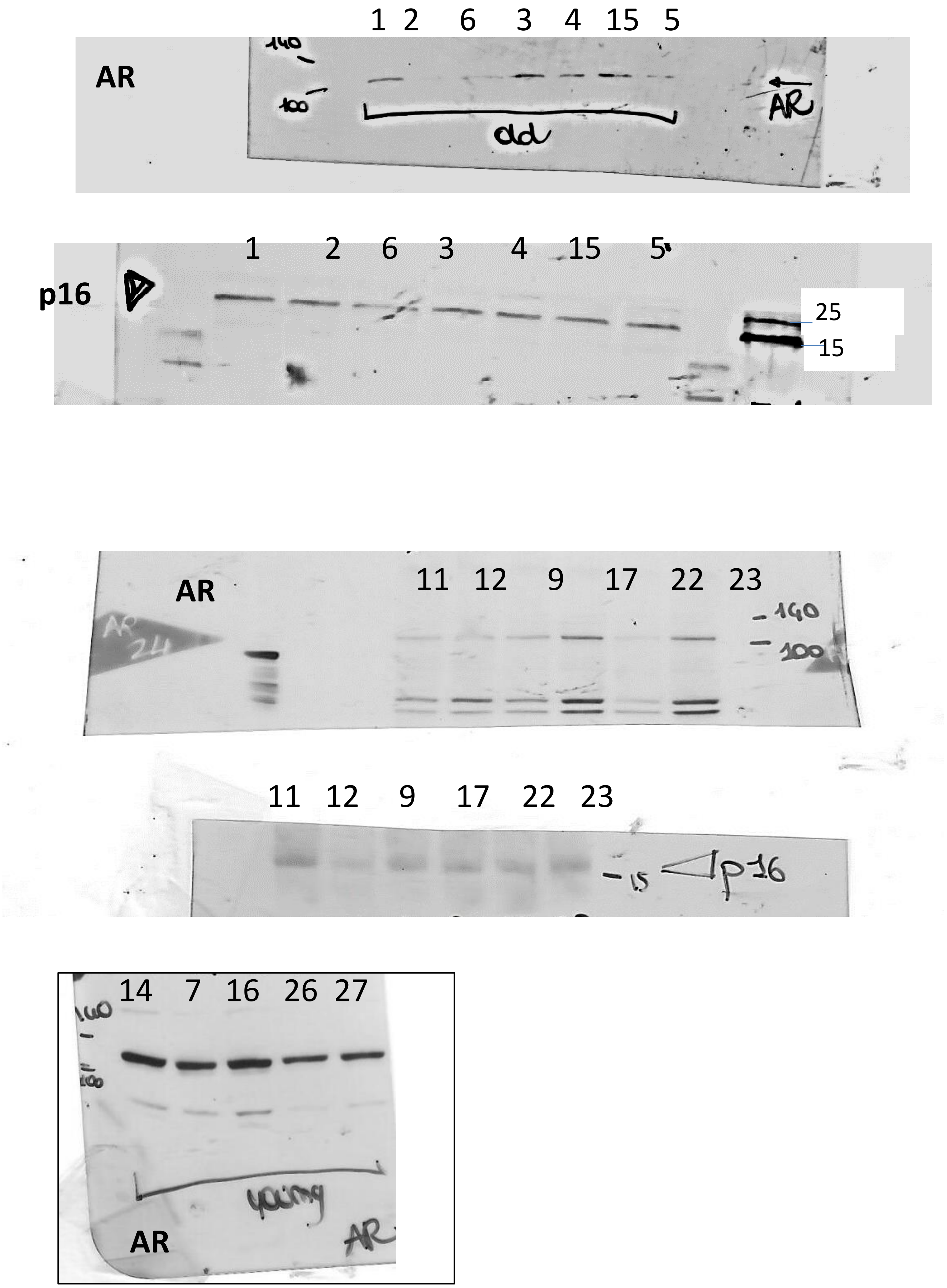

1 2 6 3 4 15 5
AR
140
100
old
AR
1 2 6 3 4 15 5
p16
25
15
11 12 9 17 22 23
AR
140
100
11 12 9 17 22 23
15
p16
14 7 16 26 27
140
100
young
AR
AR

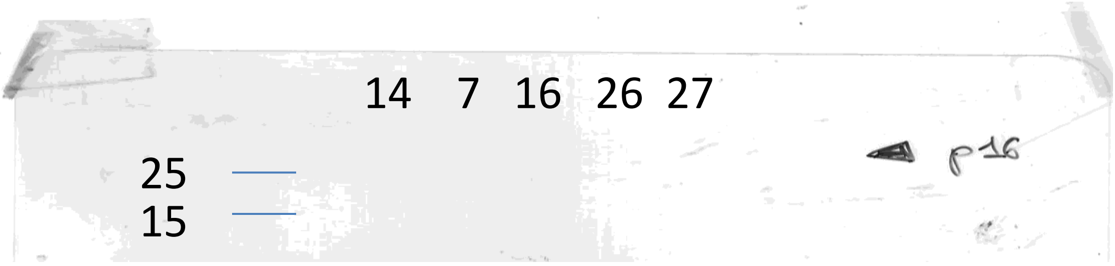
14 7 16 26 27
p16
25
15

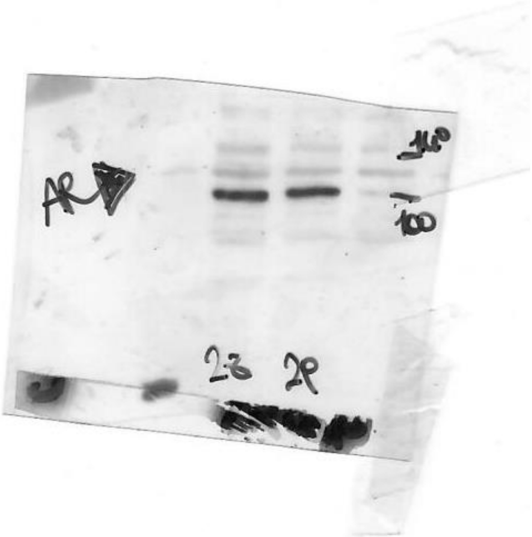
AR
100

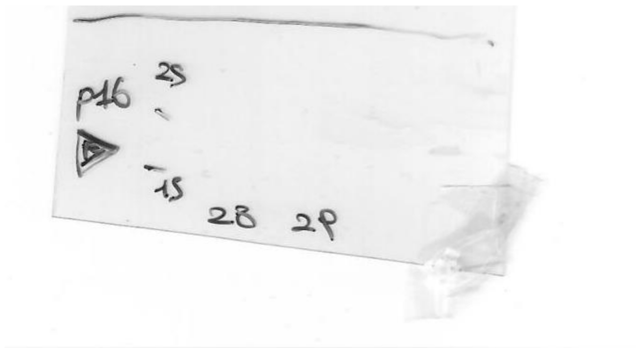
p16
25
15
28 29

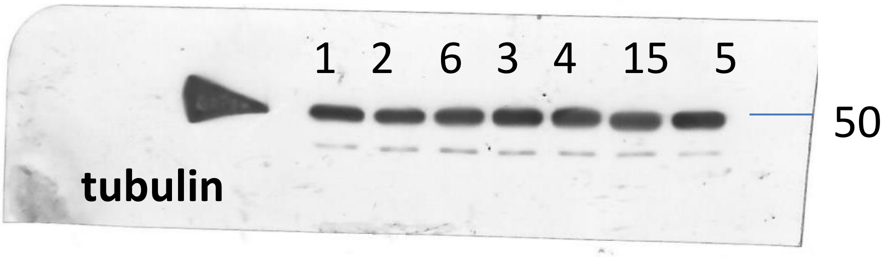
1 2 6 3 4 15 5
50
tubulin

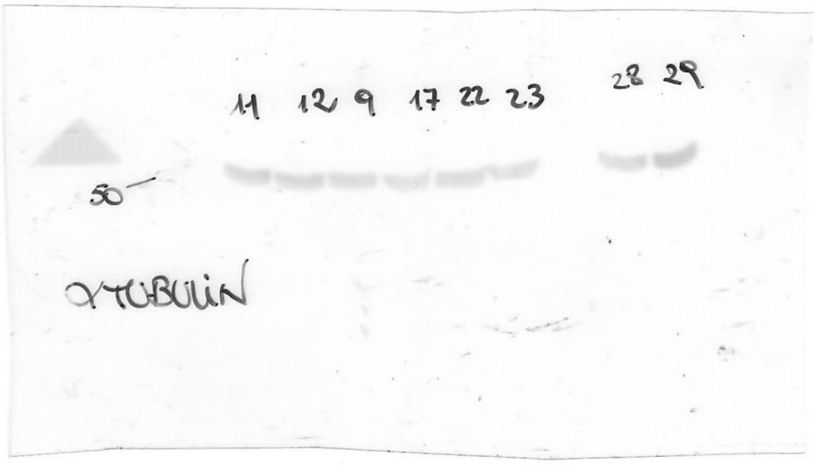
11 12 9 17 22 23 28 29
50
αTUBULIN

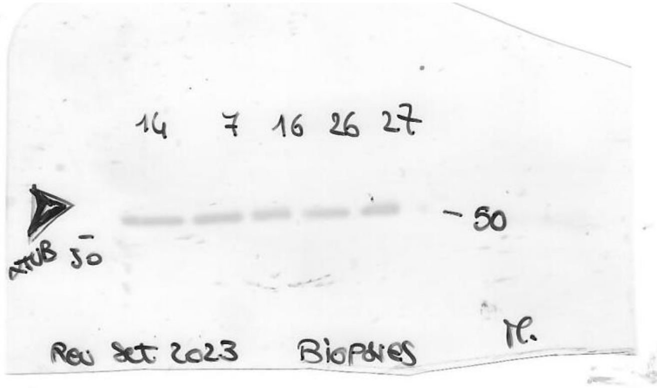
14 7 16 26 27
50
Biopsies

Figure 1 c

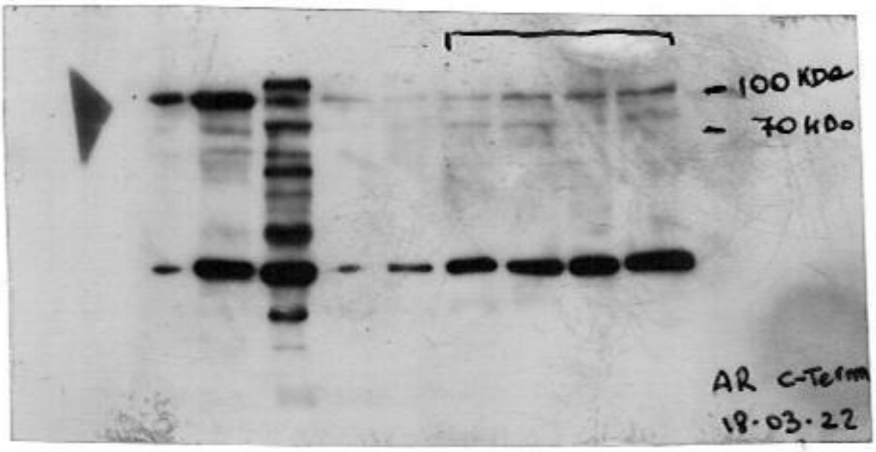

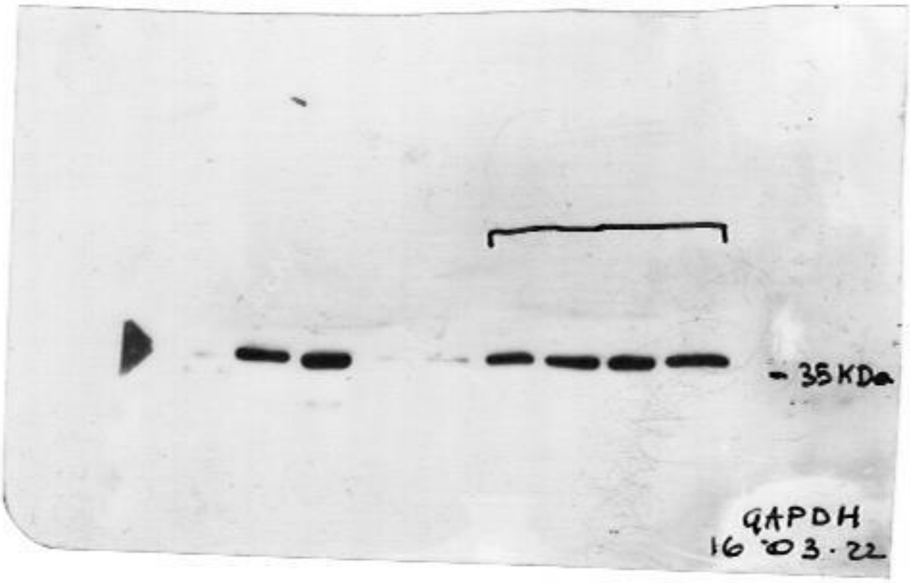

Figure 1 f

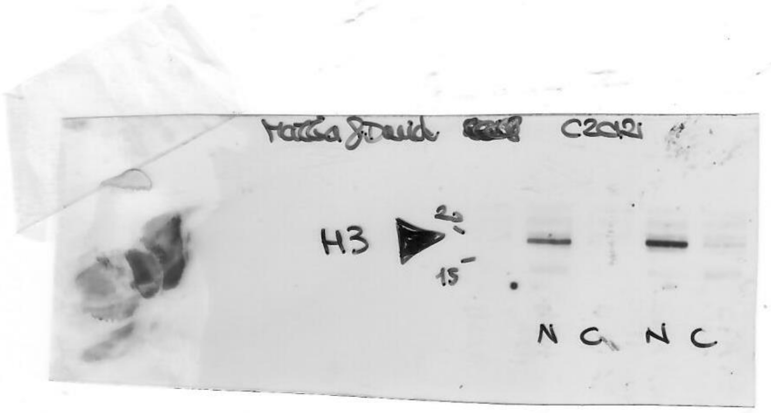
H3
20
15
N C N C

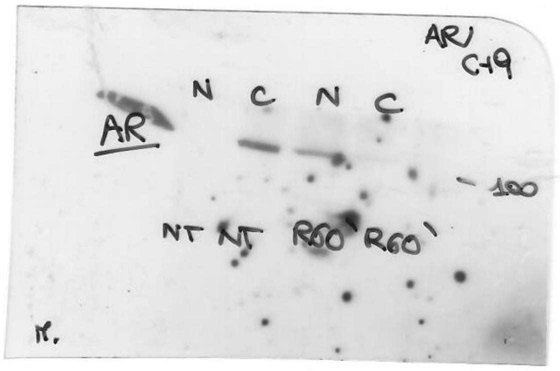
AR
N C N C
NT NT R60' R60'

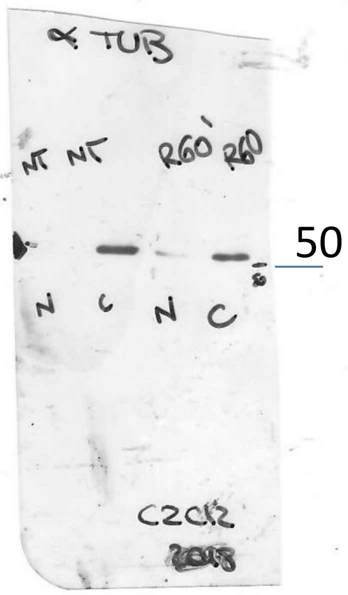
α TUB
NT NT R60' R60
N C N C
50
C2C12

Figure 2 d

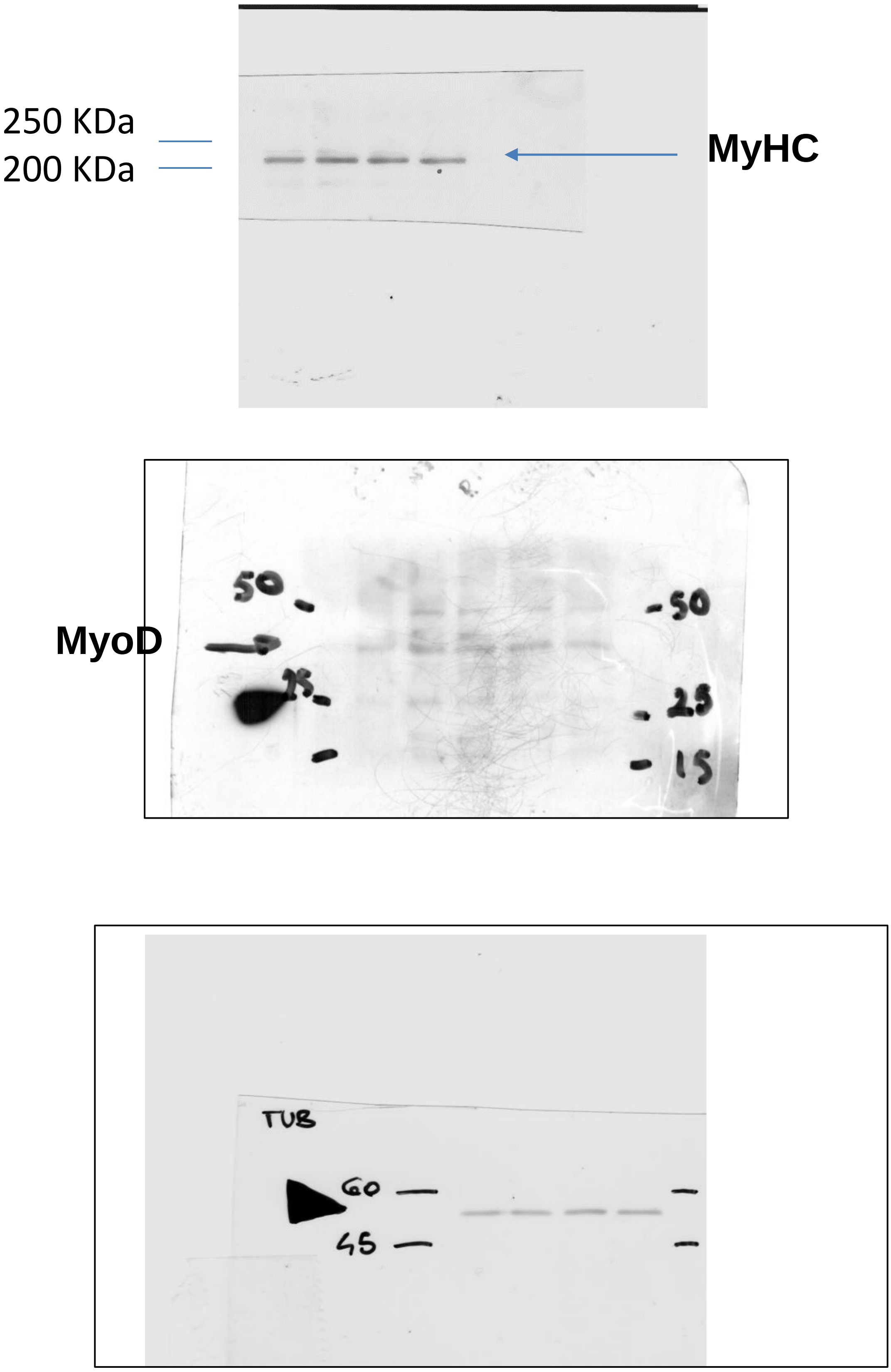

Figure 3 A

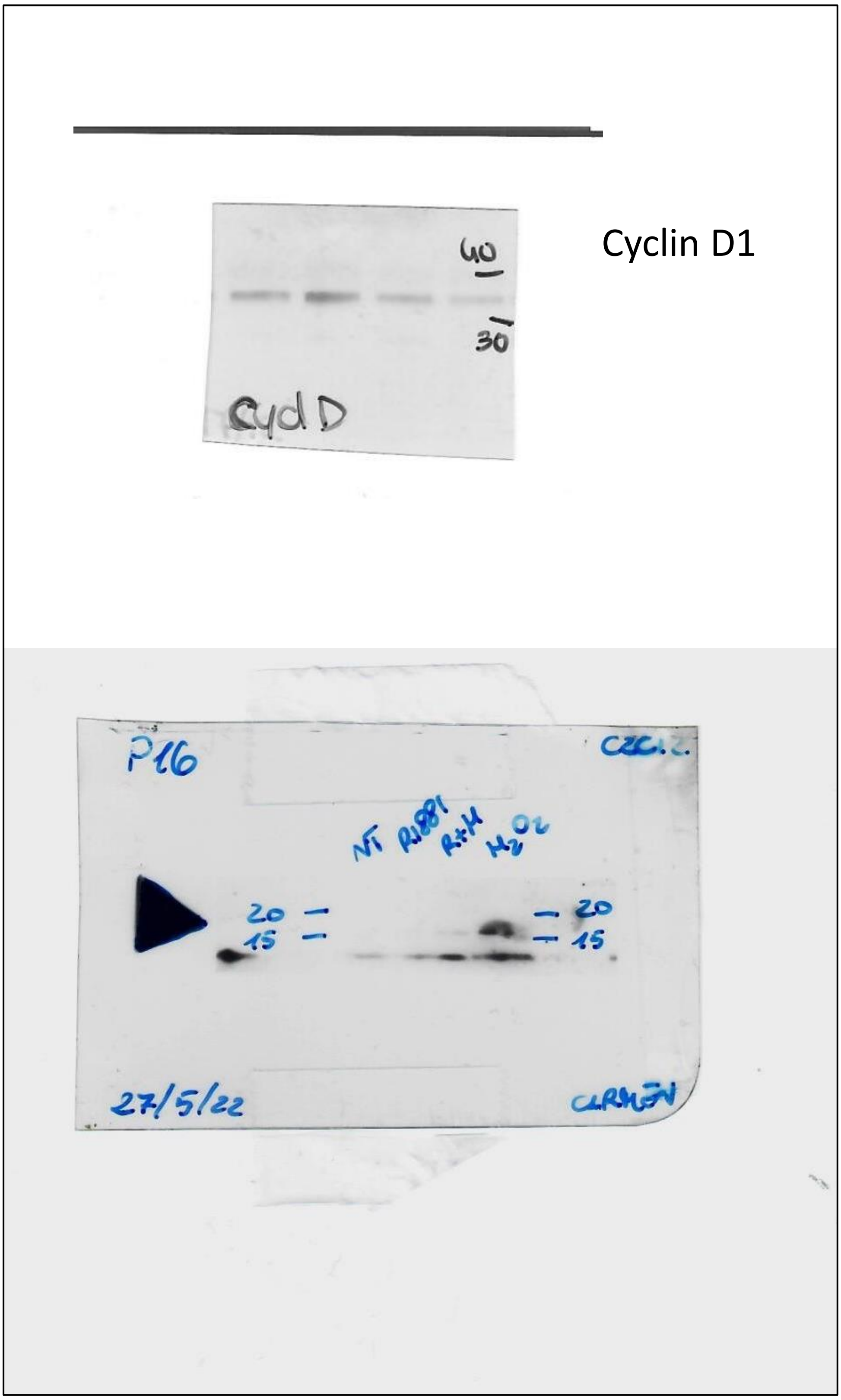

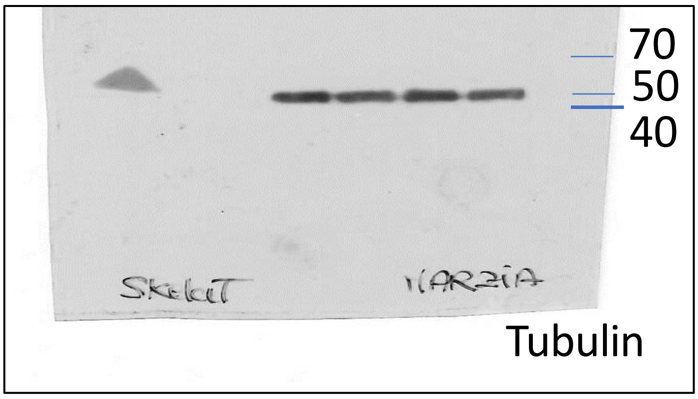

Figure 3 E

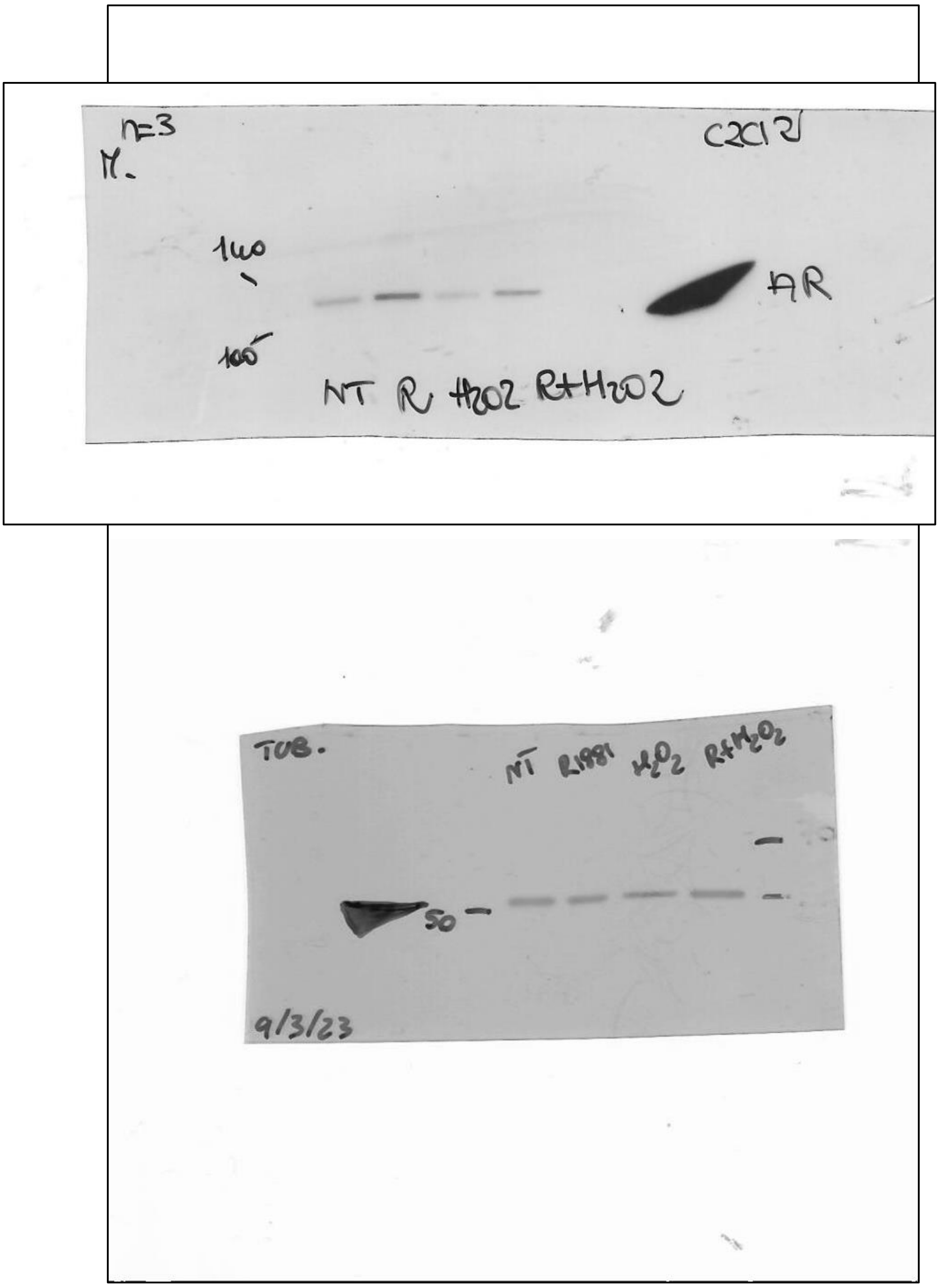
n=3
M.
C2C12
140
100
AR
NT R H2O2 R+H2O2
TUB.
NT R1881 H2O2 R+H2O2
50
9/3/23

Figure 4 a

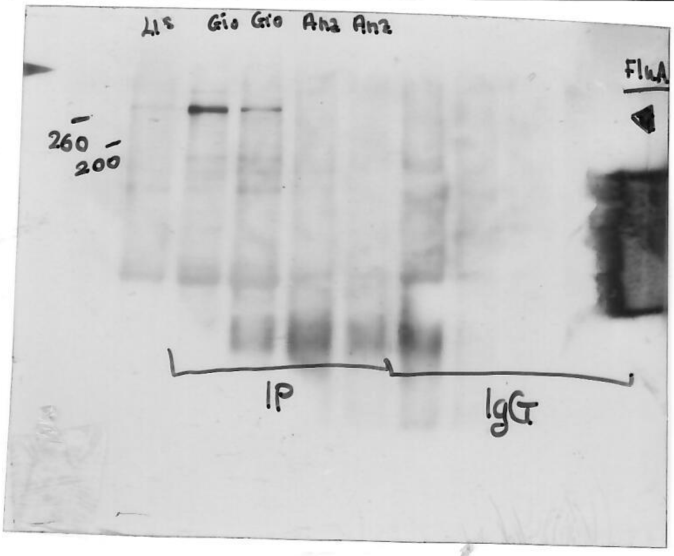
Lis Gio Gio Ana Ana
FluA
260
200
IP
IgG

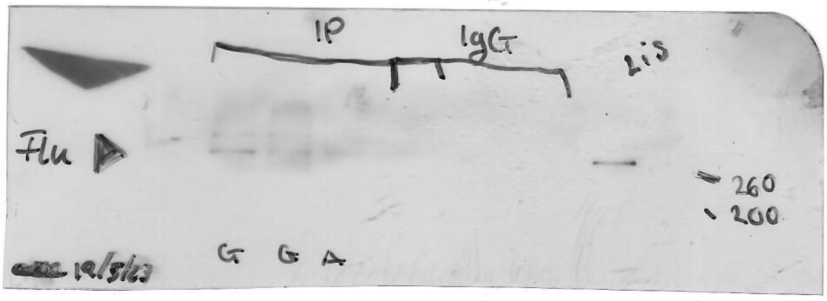
IP
IgG
Lis
Flu
260
200
G G A

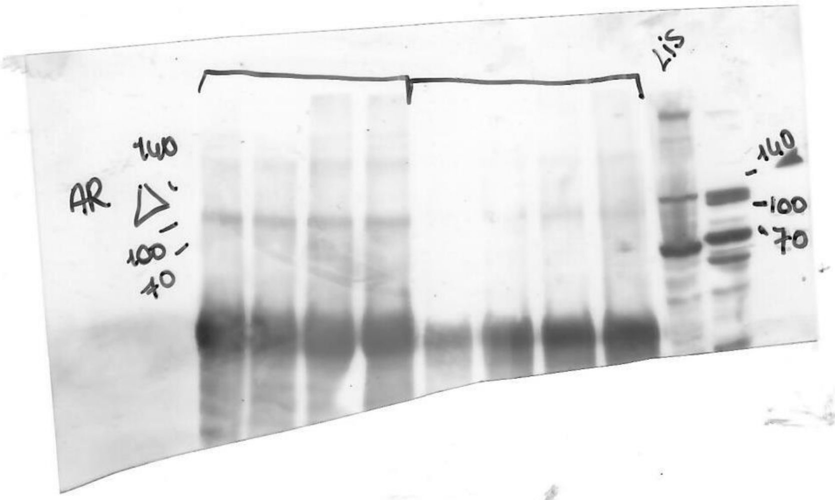
Lis
140
AR
100
40
140
100
70

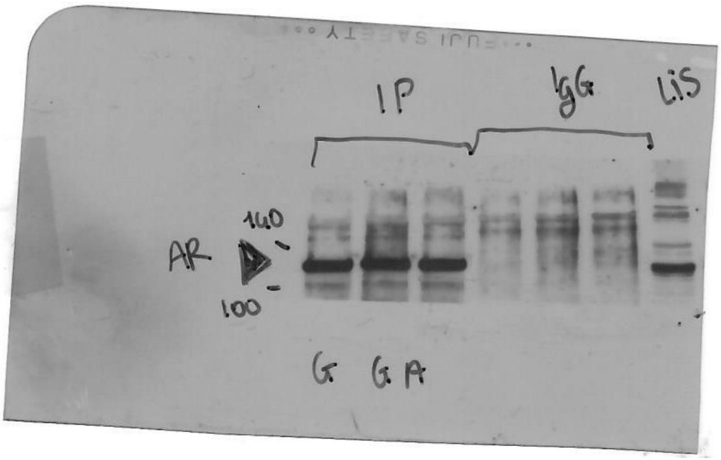
IP
IgG
LiS
140
AR
100
G G A

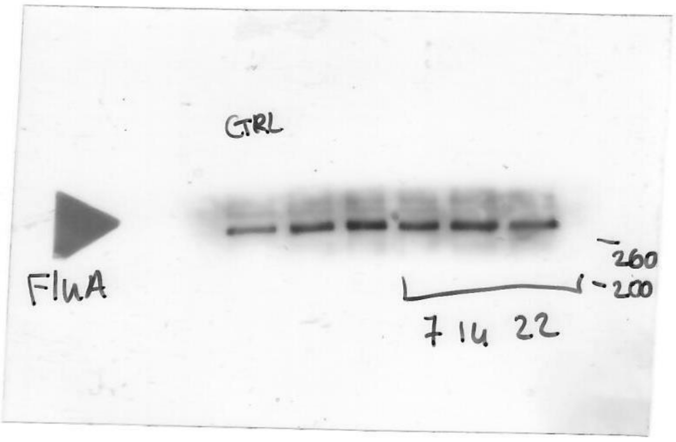
CTRL
FlnA
260
200
7 14 22

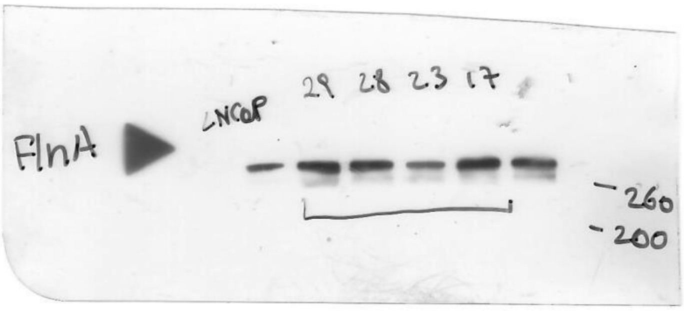
LNCaP
29 28 23 17
FlnA
260
200

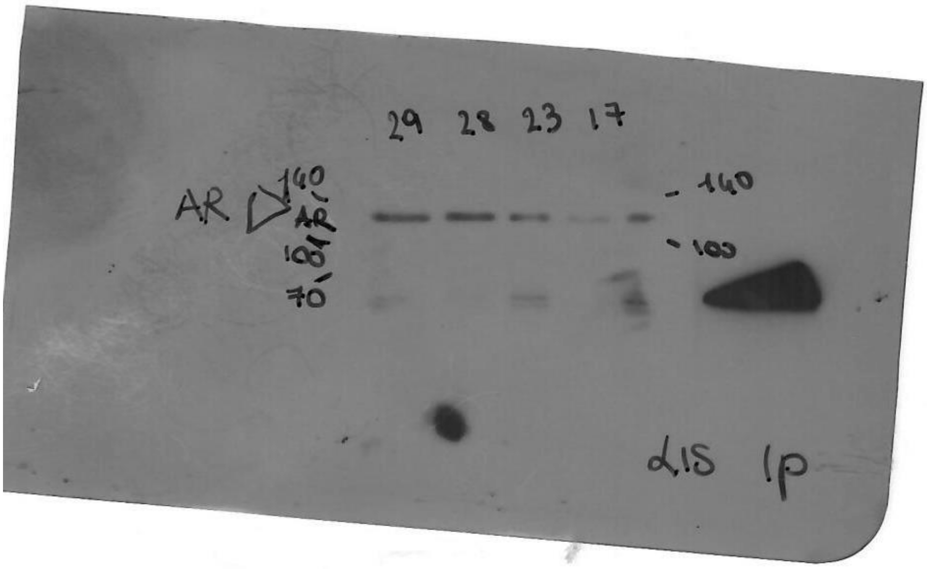
29 28 23 17
AR
140
AR
100
70
140
100

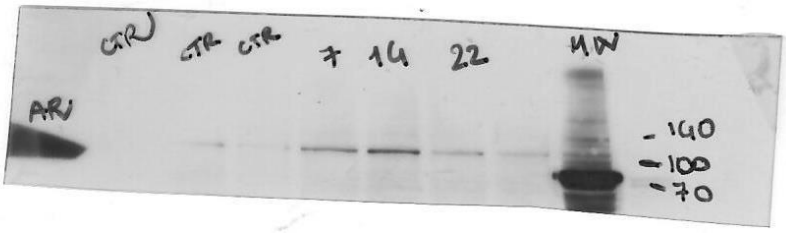
CTRL
CTR
CTR
7 14 22
MW
AR
140
100
70

Figure 4 b

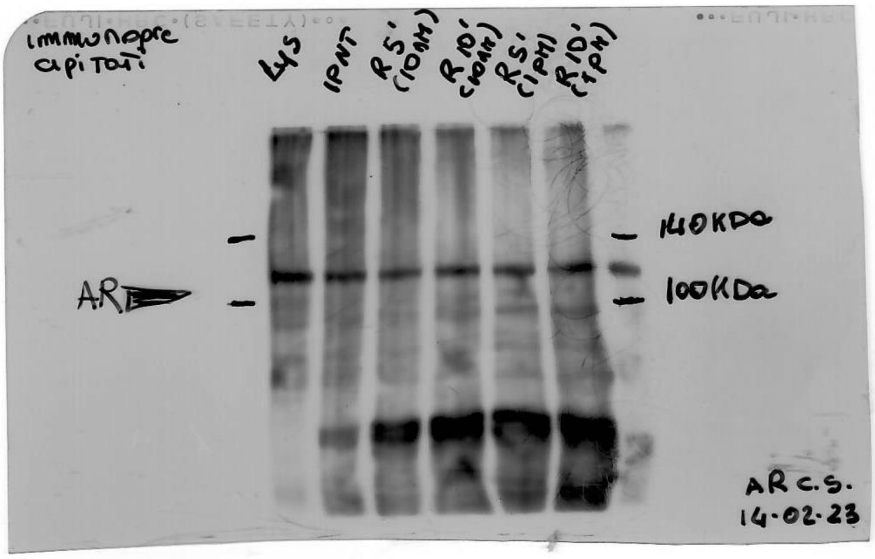
immunoprecipitati
Lys
IPNT
R5' (10nM)
R10' (10nM)
R5' (1pM)
R10' (1pM)
140KDa
100KDa
AR
AR C.S.
14-02-23

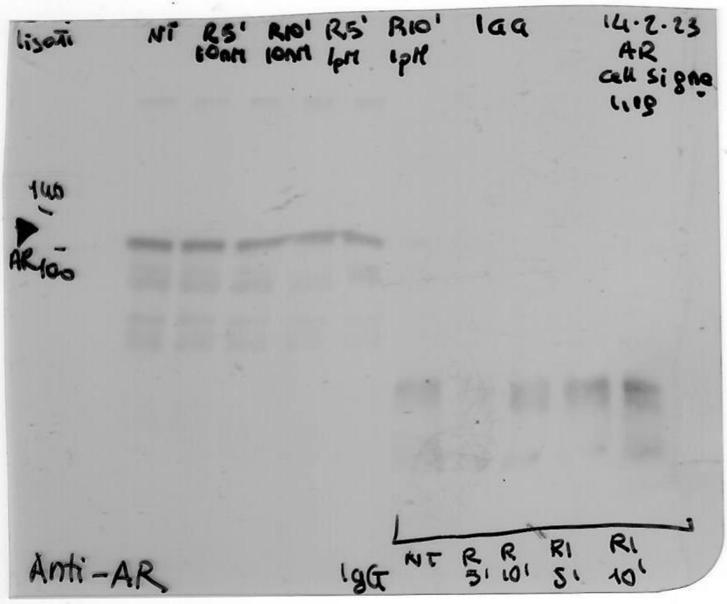
lisati
NT
R5' 10nM
R10' 10nM
R5' 1pM
R10' 1pM
IgG
14-2-23
AR
140
AR
100
Anti-AR
IgG
NT
R 5'
R 10'
R1 5'
R1 10'

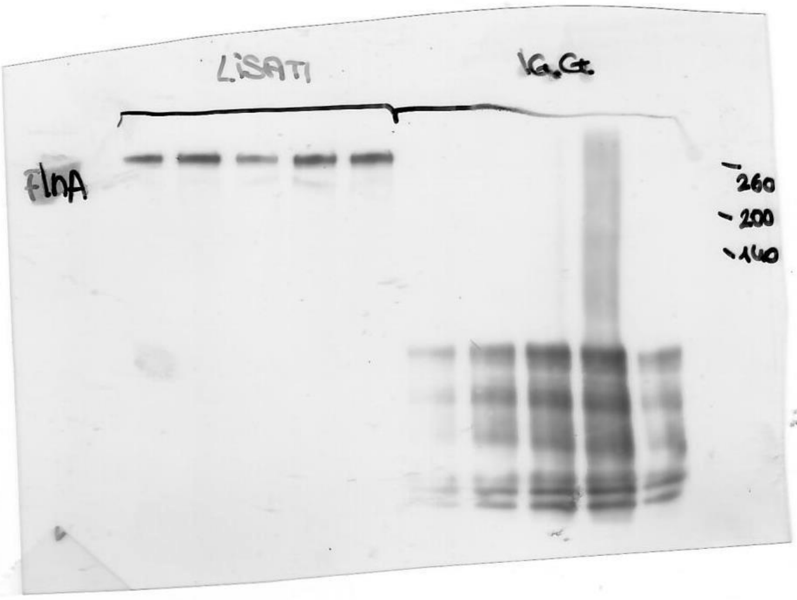
LISATI
IgG
FlnA
260
200
140

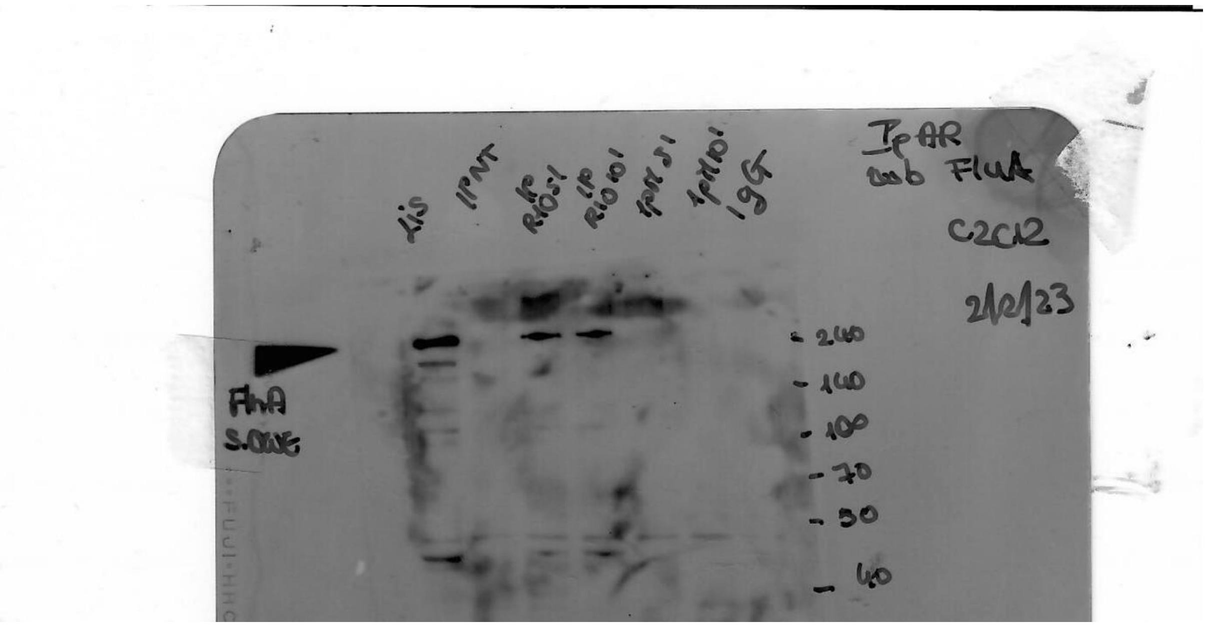
IgG
C2C12
21/2/23
FlnA
240
140
100
70
50
40

Figure 4 c

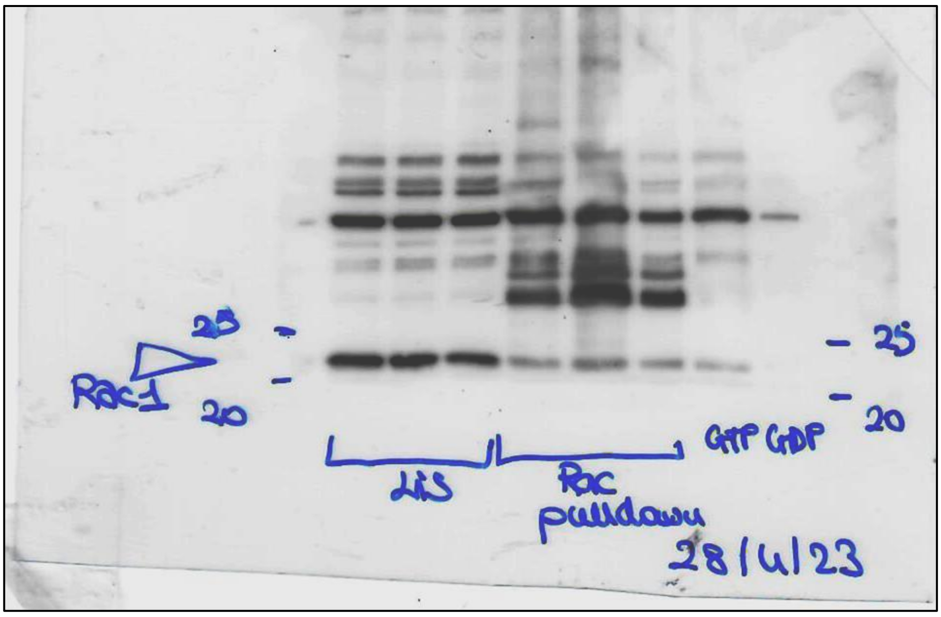
25
Rac1
20
25
20
GTP GDP
Lis
Rac
pulldown
28/4/23

Figure 4 d

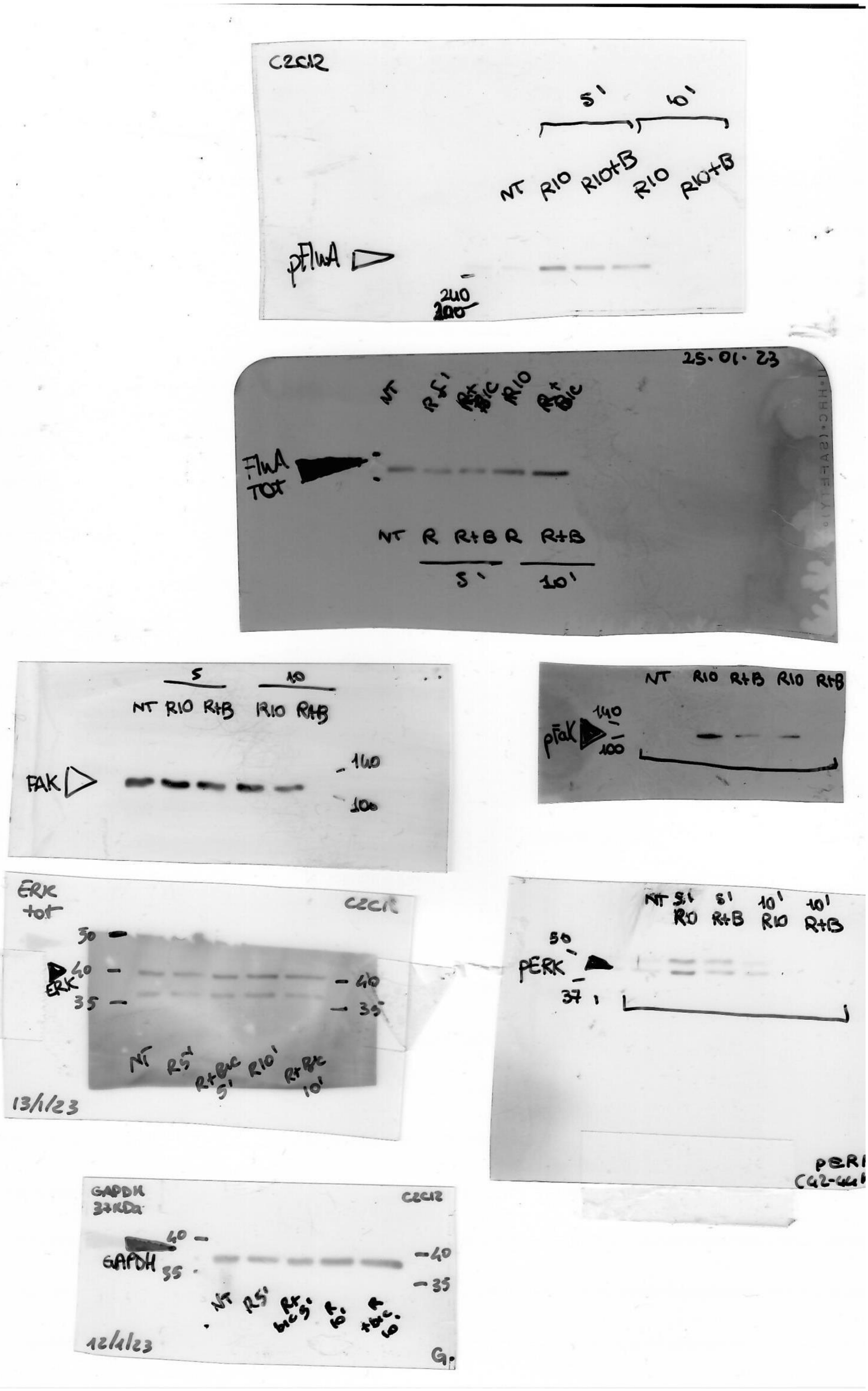
C2C12
5'
10'
NT
R10
R10+B
pFlnA
240
25.01.23
FlnA tot
R
R+B
FAK
140
100
pFAK
ERK tot
30
40
35
ERK
13/1/23
pERK
50
37
GAPDH
37kDa
12/1/23

Figure 5a

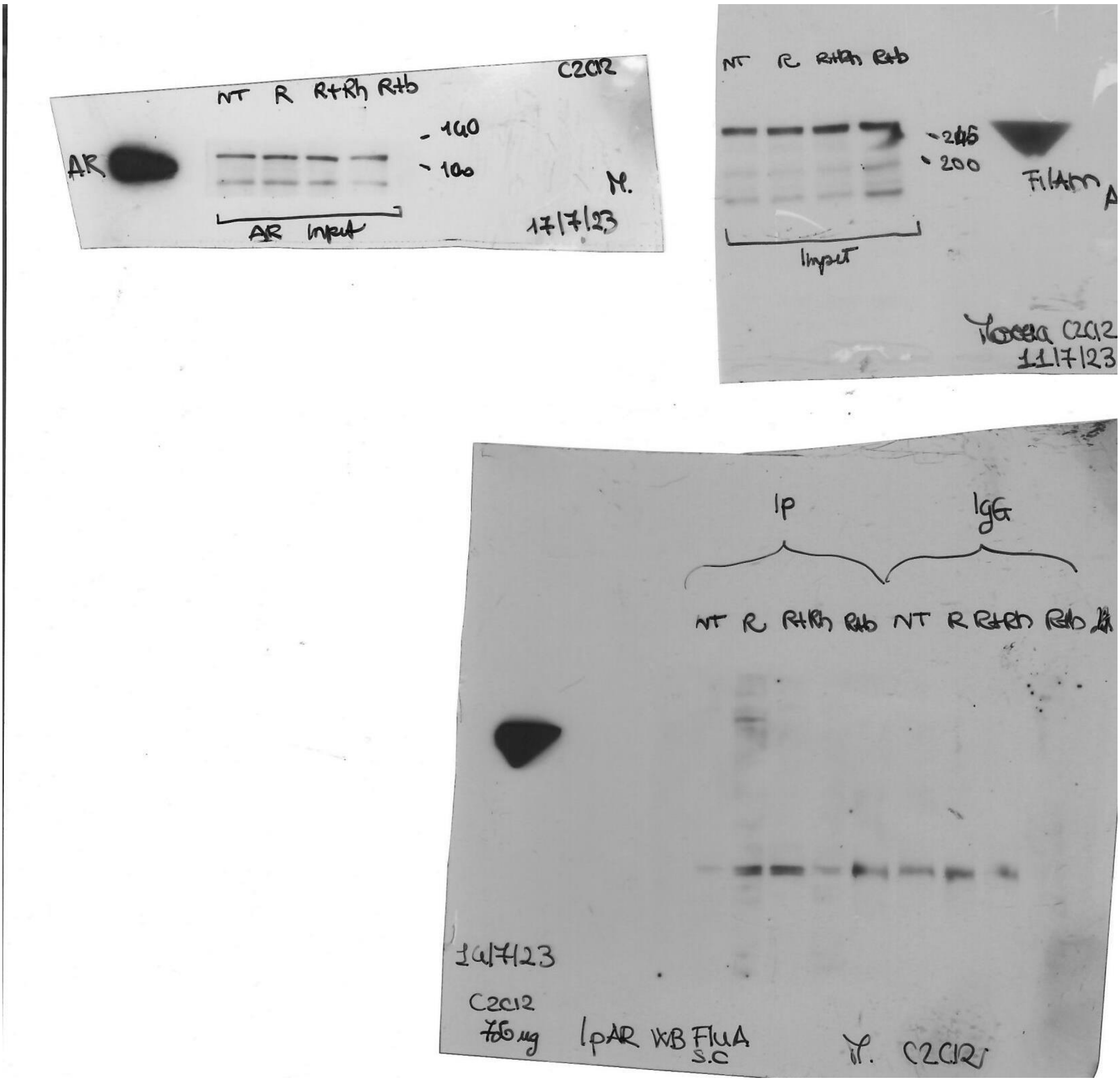
NT R R+Rh R+b
C2C12
AR
140
100
AR Input
17/7/23
NT R R+Rh R+b
245
200
FilA
Input
C2C12
11/7/23
Ip
IgG
NT R R+Rh R+b NT R R+Rh R+b
14/7/23
C2C12
IpAR WB FluA
S.C
C2C12

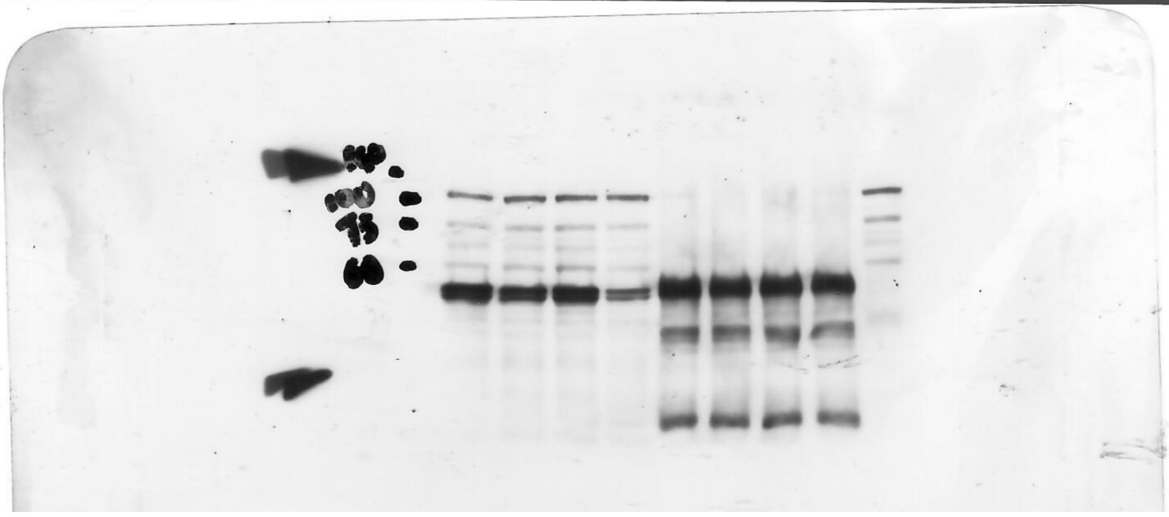
100
75
60

Figure 5b

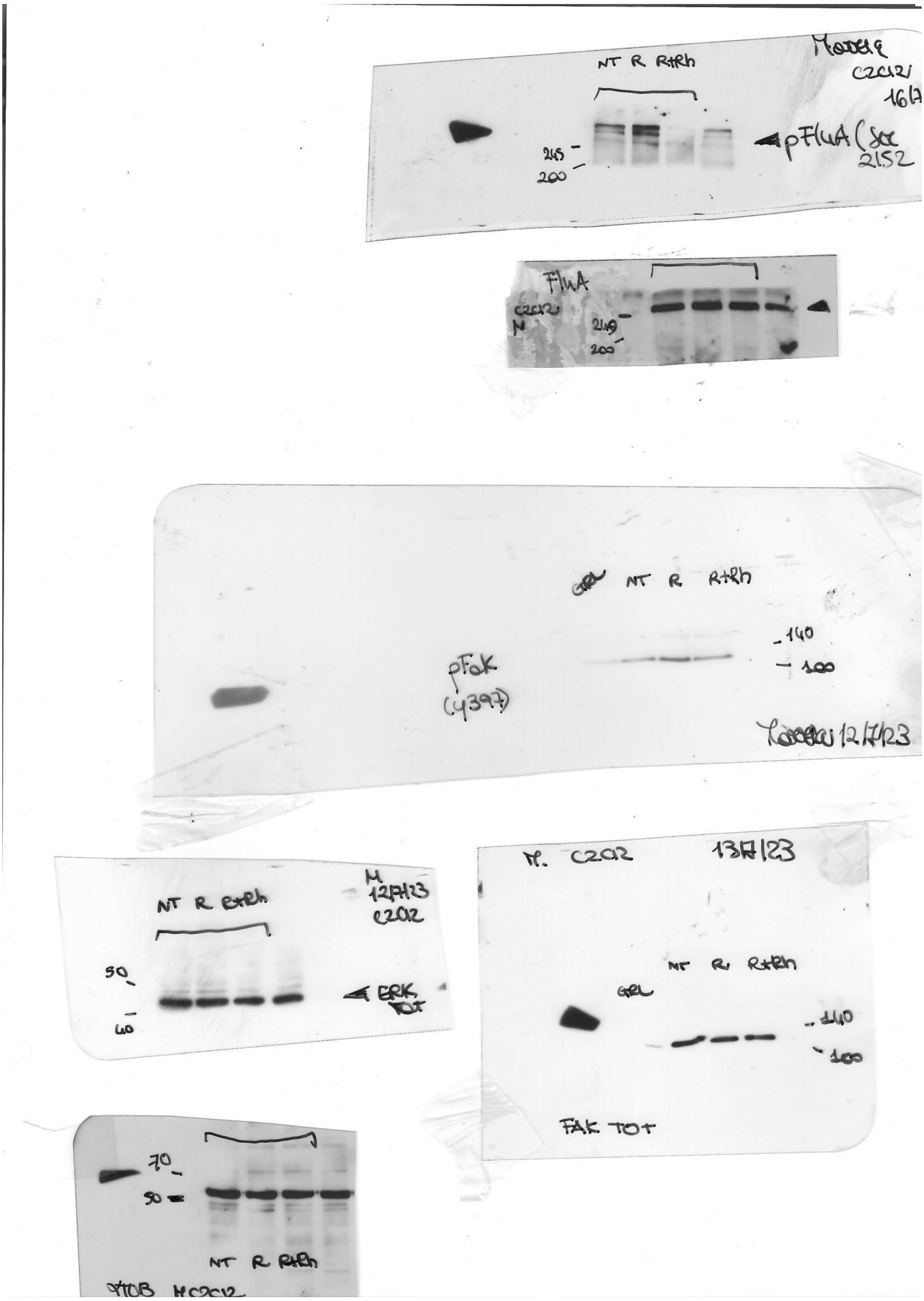
NT R R+Rh
pFlnA (Ser 2152)
245
200
FlnA
pFAK (Y397)
GRL NT R R+Rh
140
100
ERK TOT
50
40
FAK TOT
70
NT R R+Rh

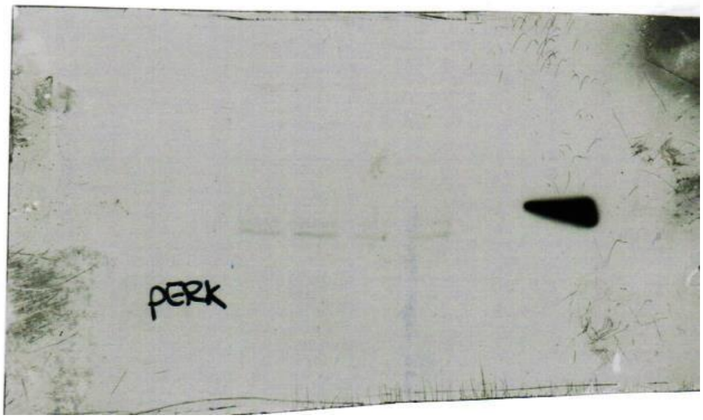
PERK

Figure 5e

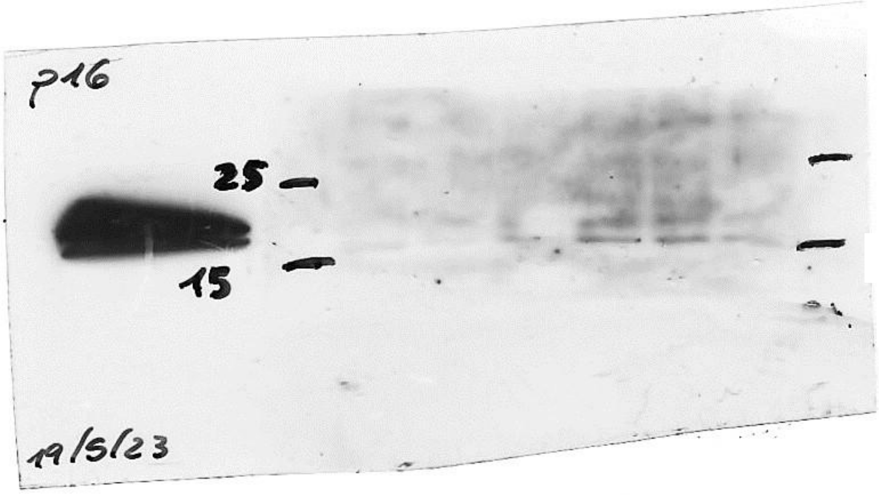
p16
25
15
19/5/23

15

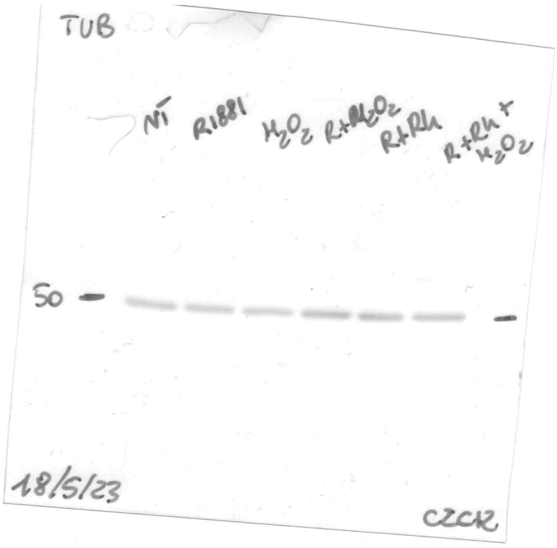
TUB
NT
R1881
H_2O_2
R+H_2O_2
R+Rh
R+Rh+ H_2O_2
50
18/5/23

Figure 1 S

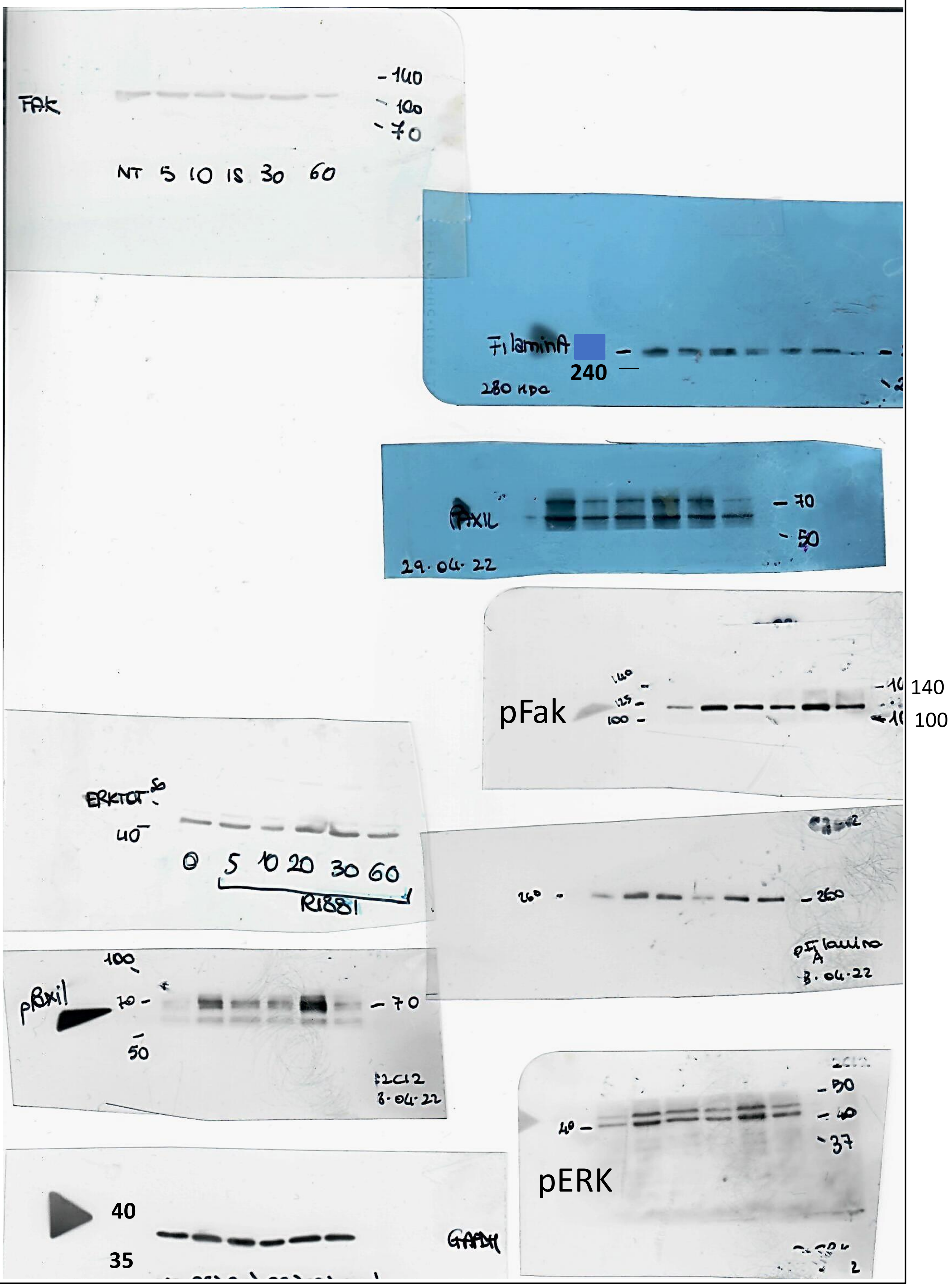
FAK
140
100
70
NT 5 10 15 30 60
FilaminA
240
280 KDa
PAXIL
70
50
29.04.22
pFak
140
100
ERKTOT
40
0 5 10 20 30 60
R1881
pPaxil
70
50
260
250
8.04.22
pERK
50
40
37
40
35
GAPDH
